# 3D Bioprinting Functional Engineered Heart Tissues

**DOI:** 10.3390/ijms262110707

**Published:** 2025-11-03

**Authors:** Man Chi Leung, Zachary Laksman

**Affiliations:** 1 Centre for Heart Lung Innovation, University of British Columbia, Vancouver, BC V6Z 1Y6, Canada; appleung@student.ubc.ca; 2 School of Biomedical Engineering, University of British Columbia, Vancouver, BC V6T 1Z3, Canada; 3 Department of Medicine, University of British Columbia, Vancouver, BC V6T 1Z8, Canada

**Keywords:** 3D bioprinting, bioinks, cardiac tissue engineering, regenerative medicine, stem cells

## Abstract

Three-dimensional (3D) bioprinting is increasingly explored as a strategy for myocardial repair and regenerative medicine. Conventional 3D casting often yields heterogeneous cellularization, slow electromechanical maturation, and inadequate vascularization; by contrast, bioprinting places cells and biomaterials in predefined architectures to program alignment, stiffness, vascular pathways, and electrical coupling that better recapitulate native myocardium. This review focuses on cardiac-specific advances in 3D bioprinting. We compare major platforms (jetting, light-based, extrusion, and volumetric) and their trade-offs for cardiac applications; distill bioink design principles trending toward natural–synthetic hybrids, including conductive and shape-morphing components; and outline practical characterization readouts spanning rheology, print fidelity, swelling/degradation, and cardiac function. We also summarize cell sources and co-culture strategies. Applications surveyed include cardiac patches, engineered tissues, chambered constructs, and organoids. Finally, we discuss current limitations and potential future directions for 3D bioprinting cardiac tissues. Collectively, recent advances position 3D bioprinting to accelerate the realization of in vivo-like engineered heart tissues.

## 1. Introduction

Regenerative medicine is central to cardiology because the adult heart has limited intrinsic repair: after myocardial infarction, lost myocardium is replaced by non-contractile scar, degrading pump function [1]. To restore function—or to model disease credibly in vitro—we need tissues that recapitulate cardiac architecture, electromechanics, and vascular support [2].

3D bioprinting addresses this need by depositing cells and biomaterials in predefined patterns, so alignment, stiffness, vasculature, and even conduction paths can be directly programmed into the construct [2,3]. Compared with conventional 2D monolayers and 3D casting, it offers higher spatial control, better reproducibility, and straightforward multi-material/multi-cell patterning—advantages for both in vitro testing (more predictive maturity, standardized readouts) and translational grafts (patient-specific geometry, guided integration) [4].

The field is trending from extrusion-based printing toward higher-resolution or faster modalities (jetting, light-based, and volumetric) [5,6,7]. Bioinks are shifting from single natural polymers to hybrids that combine bioactivity (dECM, collagen, fibrin) with synthetic tunability (GelMA, PEG, MeHA) and, where needed, conductive or shape-morphing components [8,9,10]. Scale is expanding from microtissues and strips to chambered constructs and organoids [11,12].

In this review, we will provide a concise overview of 3D bioprinting modalities and their trade-offs; summarize current bioink strategies and testing standards (rheology, fidelity, viability, force/conduction); and outline cell-source choices and co-culture tactics that stabilize function and vascularization. Throughout, we emphasize recent developments and provide guidance for teams adopting 3D bioprinting in cardiac regenerative medicine—whether the goal is robust in vitro models or implantable tissues.

## 2. Three-Dimensional Bioprinting in Cardiac Tissue Engineering

Three-dimensional (3D) bioprinting has emerged as a transformative technology for fabricating complex tissue architectures through the layer-by-layer deposition of biomaterials, living cells, and bioactive molecules [2,13]. Unlike conventional additive manufacturing, which focuses primarily on shaping inert materials, 3D bioprinting in regenerative medicine requires bioinks with high biocompatibility to ensure proper integration of functional cells and signaling molecules [3]. This capability provides precise control over cell composition, spatial distribution, and tissue morphology [3]. While it is important to note that some modalities or biomaterials may pose toxicity problems to the cells, printing the ink in a patterned way and seeding the cells after still prove to improve cell maturation compared to earlier 3D processing strategies including casting or spheroid/organoid formats [4,14].

Before printing, the process begins with a digital design stage, where computer-aided design (CAD) tools and computer numerical control (CNC) systems generate coordinate files (e.g., g-code) that guide the bioprinter [15,16,17]. This integration of design and fabrication ensures fidelity and reproducibility in constructing complex tissue geometries [18].

During the printing process, key parameters—such as nozzle diameter and extrusion speed in extrusion-based systems, or light intensity in light-based platforms—must be carefully optimized alongside temperature, layer thickness, and number of layers. Fine-tuning these variables is essential to maintain cell viability and construct fidelity, as they directly influence both mechanical integrity and biological function [16,19].

After printing, constructs undergo comprehensive evaluation of their structural and functional properties. Standard assays assess shape fidelity, mechanical stability, elasticity, swelling, degradation, and cell–material interactions [19]. For cardiac applications, additional analyses are performed to probe cardiac-specific functionality, including biomarker expression, contractile force generation, electrophysiological activity, and calcium handling. These post-fabrication assessments are critical for refining the bioprinting process and ensuring that engineered tissues exhibit structural and functional properties comparable to native myocardium [15,20].

## 3. Bioprinting Techniques

3D bioprinting techniques used in cardiac tissue engineering (CTE) can be categorized into four main types, including jetting-based, stereolithography- and digital light processing–based, extrusion-based, and volumetric bioprinting, each possessing distinct advantages and limitations. A summary table of the characteristics of different printing techniques, including resolution, bioink viscosity range, and cell concentration range, is provided (Table 1).

### 3.1. Jestting-Based Bioprinting

**Figure 1:**
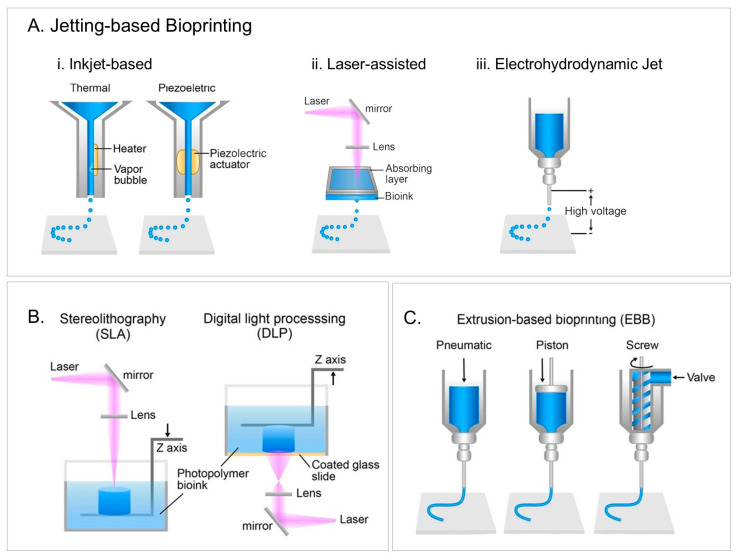
Schematic of three bioprinting modalities: (**A**) jetting-based; (**B**) stereolithography (SLA) and digital light processing (DLP); (**C**) extrusion-based. (Figure reproduced with permission from Pu et al. [22]).

Jetting-based bioprinting, or droplet-based bioprinting, uses a drop-on-demand strategy. It enables the precise, contactless deposition of cells and bioactive materials as droplets to fabricate 3D tissues or organs. It offers fine control over cell density by adjusting the number of cell-laden droplets deposited at specific locations [21]. Some commonly used examples of jetting-based bioprinting in the field of regenerative medicine include inkjet-based, laser-assisted and electrohydrodynamic jet bioprinting (Figure 1A). The following subsections introduce the major types of jetting-based bioprinting.

#### 3.1.1. Inkjet-Based Bioprinting

Inkjet bioprinting generates droplets via controlled thermal or piezoelectric mechanisms. Thermal systems typically eject ~10–100 pL droplets (≈20–100 µm diameter), while piezoelectric systems span ~1–300 pL (≈30–100 µm diameter) [21]. These small droplet volumes enable high resolution and precise, programmable cell placement. Because inkjet printing relies more on waveform control than fixed hardware, designs are flexible and easily modified; multiple bioinks and cell types can be interchanged within a single run [23]. However, reliable droplet formation generally requires low-viscosity bioinks (~3–10 mPa·s), which narrows material choices for cardiac applications. Additional issues include nozzle clogging from cell/biomolecule aggregation and satellite droplets that cause non-uniform deposition and poorer print quality [21]. Recent advances have addressed these limitations. Zhu et al. [24] engineered a “multi-physical” printhead that warms the nozzle, chills the air just ahead of it, and cures each landing drop with UV; this allows inks roughly three times more viscous than usual to flow, jet, and solidify in one step, effectively widening the usable viscosity window. Yang et al. [25] introduced a multi-pulse actuation waveform that cancels pressure echoes from the initial pulse so each cycle yields a single, uniform droplet—stabilizing droplet size and shape across the print. While these are impressive innovations, the suitability of inkjet and these upgrades for cardiac tissue printing still requires thorough evaluation and validation.

#### 3.1.2. Laser-Assisted Bioprinting

Laser-assisted bioprinting (LAB), or laser-induced forward transfer (LIFT), consists of a donor substrate with an energy-absorbing layer, a collector slide and a focused pulsed laser beam. The energy-absorbing layer, which is often a thin metallic film, is coated with a cell-laden bioink. When excited by a focused laser beam, the laser vaporizes a minute volume of the metal, generating a force that propels a controlled droplet onto a collector slide [21]. Studies have shown that >95% cell viability can be maintained when pulsing energy is kept within the range of 1–20 μJ [26]. The droplet size ranges from 50 to 500 µm, which is slightly larger than inkjet bioprinting. However, because the process is nozzle-free and chamber-free, it avoids clogging issues and prevents shear stress on cells caused by nozzle-orifice restrictions. As the bioinks are not enclosed in a chamber, LIFT also allows a wider range of printable bioinks with varying viscosities (Table 1). Because LIFT offers finer resolution, it can fabricate more complex and detailed constructs. For example, Ji et al. [27] used the Lumen X laser-based bioprinter to print induced pluripotent stem cells (iPSCs) into highly branched 3D Purkinje structures. The study showed high reproducibility (100% success rate in structure generation) and highlighted the potential of LIFT for printing cardiac cells without compromising viability under transient laser-induced heating.

#### 3.1.3. Electrohydrodynamic Jet

Electrohydrodynamic jet (EHD) bioprinting deposits cell-laden droplets by generating an electric field between the nozzle and substrate and thereby pulling the droplets toward the substrate. Bioink deposition is controlled by substrate positioning and droplet ejection. EHD can achieve higher resolution due to the use of a small nozzle orifice, and lateral variations in droplets can be minimized by the focused electric field [21]. A study by Mao et al. [14] bioprinted a fibrin-based anisotropic, microfibrous lattice scaffold using a melt-based EHD and for seeded cardiomyocytes (CMs). With this printing technique, the scaffold consisted of interconnected longitudinal pores and staggered transverse fibers, mimicking the fiber orientation of native myocardium and promoting uniaxial CM alignment. Critical considerations apply to both bioink properties and post-printing steps. The EHD printing process depends strongly on bioink’s surface tension, viscosity, electrical conductivity and evaporation rate, necessitating careful selection to ensure printability. Additionally, due to the use of electric fields, charge can accumulate within printed fibers, leading to disordered fiber arrangement—a critical concern for maintaining anisotropy and avoiding undesired conduction triggering in cardiac tissues.

Jetting-based bioprinting offers contactless, precise spatial deposition of cells and biomaterials. While inkjet bioprinting shows the possibility to print and switch between different cell types in one print, laser-assisted bioprinting enables complex structures such as Purkinje fibers owing to its nozzle-free and chamber-free operation, and EHD achieves high resolution. However, relatively few studies in cardiac tissue engineering (CTE) have been reported, likely for several reasons. One limitation is thickness: jetting-based prints typically produce constructs ~50–500 µm thick [14,26,27], whereas native cardiac tissue is roughly an order of magnitude thicker [28], and which is challenging to achieve with droplet-based methods. Another factor is limited commercial availability; for example, the Lumen X used in Ji et al. [27] has only recently become commercially available and optimized. Printing parameters in jetting-based systems—such as laser intensity, temperature, and bioink deposition—are not standardized and require extensive optimization. Further investigation is needed into feasibility, particularly bioink availability/selection and cell viability. Overall, jetting-based bioprinting remains an efficient and high-resolution option for specific applications in CTE—such as the complex conduction system models—under careful considerations on the bioink selection, cytocompatibility and print parameters optimization.

### 3.2. Stereolithography and Digital Light Processing (DLP)

Stereolithography and digital light processing (DLP) bioprinting are both light-based fabrication techniques that rely on polymerizing photocrosslinkable bioinks to create 3D constructs. In stereolithography, a UV laser selectively cures a thin layer of photopolymer resin within a tank. The build platform lowers to form a thin film of liquid resin, which is then solidified layer by layer as the laser traces the desired pattern point by point (Figure 1B). A key advantage of stereolithography is that, by varying printing parameters such as laser attenuation, laser intensity, or scanning speed, it is possible to create graded crosslinking—i.e., some regions are more crosslinked while others are less. This mismatch generates internal stress that drives 4D morphing effects [29]. Using this strategy, Cui et al. [29] fabricated a cardiac patch that autonomously curves to match the heart’s shape. With stereolithography, they also produced helically arranged myocardial fibers with precisely controlled angles to replicate the anisotropy of native myocardium. A limitation is speed: the laser must scan the entire surface point by point, which can be time-consuming. An innovative study by Miao et al. [30] integrated photolithography with stereolithography so that photolithography polymerizes an entire surface in seconds through a photomask, while leaving a ~10 µm liquid layer for fine fabrication to selectively cure the top film into microgrooves. This design completes prints within ~15 min while maintaining fidelity and patterning.

DLP uses a digital light projector and a digital micromirror device (DMD) to cure an entire layer of photocurable bioink simultaneously with UV light, then advances to the next layer (Figure 1B). This eliminates x–y mechanical scanning during curing and reduces print time [31]. Despite this advantage, axial resolution can be compromised due to discretization from mask changes between layers [32]. A notable advance is microscale continuous optical printing (µCOP), which fabricates complex microarchitectures continuously by moving the stage smoothly along the *z*-axis while dynamically updating the mask [33]. Liu et al. [33] also demonstrated multi-material printing—e.g., a cantilever for force analysis and a cardiac cell-laden bioink—using DLP without additional post-processing, enabling seamless transitions between materials.

Fewer studies encapsulate cells directly in the ink for stereolithography because photoinitiators and UV exposure pose cytotoxicity risks, including potential DNA damage [29,34]. As a result, many groups print acellular scaffolds and seed cells afterward, which can lead to non-uniform cell distribution [29]. DLP is generally associated with lower cytotoxicity risk [32,33], and several studies have printed cell-laden constructs with ~85–90% viability, likely due to lower local light intensity and shorter exposure times.

### 3.3. Extrusion-Based Bioprinting

Extrusion-based bioprinting is a widely used technique in CTE that deposits bioinks—typically viscoelastic, cell-laden materials—through one or more nozzles in a computer-controlled, layer-by-layer process [5]. Pneumatic pressure or mechanical force (piston- or screw-driven) is applied to extrude the bioink as cylindrical filaments onto a build platform (Figure 1C). This approach is popular for cardiac tissues because shear stresses within the nozzle, along with extensional stresses during extrusion, can align microfibers in the bioink in a preferred direction [35,36,37], thereby guiding cardiomyocyte alignment. Recreating this anisotropy is a critical and desirable feature in cardiac constructs. Extrusion-based printing also allows straightforward parameter tuning—pressure, nozzle diameter and geometry, stand-off distance, and filament thickness—to mimic characteristics of native myocardium [11]. It is also relatively simple and cost-effective.

**Figure 2:**
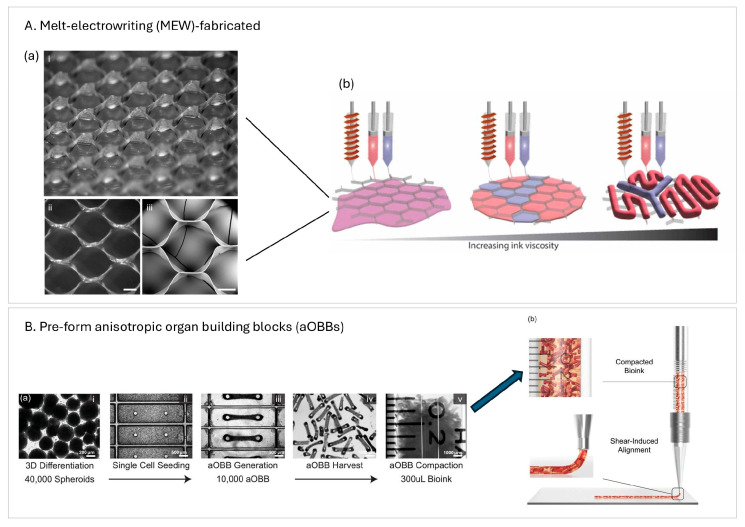
Upstream manufacturing strategies prior to extrusion-based bioprinting. Study 1—MEW PCL meshes. (**A**) Workflow overview. ((**a**), **i**–**iii**) Hexagonal melt-electrowritten (MEW) poly(ε-caprolactone) (PCL) microfiber meshes imaged by stereomicroscopy (**i**,**ii**) and scanning electron microscopy (**iii**); scale bars, 250 µm. (**b**) Schematic of extruding hydrogels with varied polymer/crosslink density into the MEW mesh. Study 2—anisotropic organ building blocks (aOBBs). (**B**) Workflow overview. (**a**) Preparation of an extrudable bioink: cardiac spheroids are generated (**i**) and dissociated to cardiomyocytes (**ii**), mixed with fibroblasts (9:1), suspended in a collagen-based gel, seeded into micropillar arrays to self-assemble into aOBBs (**iii**), harvested (**iv**), and compacted (**v**). Scale bars: (**i**) 200 µm; (**ii**,**iii**) 500 µm; and (**iii**,**iv**) 1000 µm. (**b**) aOBBs collected into a syringe for subsequent extrusion. ((**A**) reproduced with permission from Ainsworth et al. [6]; (**B**) reproduced with permission from Ahrens et al. [38].).

Two recent studies highlight the strategic use of pre-processing and auxiliary fabrication steps to enhance structural and functional outcomes. In one, melt-electrowriting (MEW) was used to fabricate hexagonal PCL microfiber scaffolds prior to extruding cell-laden bioinks [6] (Figure 2A). The resulting rigid, reinforcing scaffold provided mechanical support, promoted anisotropic cell alignment, and enabled integration of a patterned pre-vascular network—addressing limitations of soft hydrogel-only constructs and facilitating vascularization upon implantation. In another, the authors pre-formed anisotropic organ building blocks (aOBBs) on PDMS micropillar arrays before bioprinting, yielding tissues with elongated, microscale alignment and improved electromechanical maturation prior to encapsulation (Figure 2B); the additional shear and extensional stresses during extrusion further enhanced alignment [38]. As a result, aOBB-printed tissues exhibited significantly higher contractile force and more organized function while using fewer cells. Together, these studies also illustrate how integrated pre-fabrication strategies can streamline multicellular bioprinting workflows and improve tissue performance.

#### Suspended/Embedding Bioprinting

**Figure 3:**
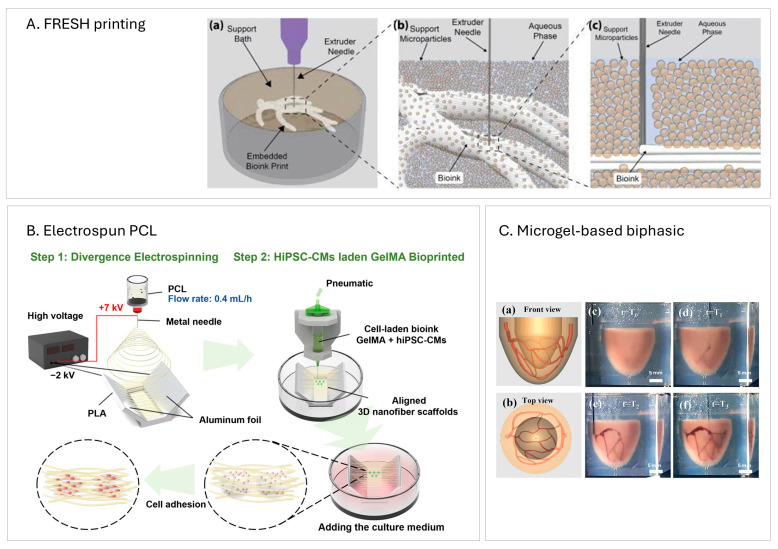
Suspended/embedded extrusion bioprinting strategies. (**A**) Freeform Reversible Embedding of Suspended Hydrogels (FRESH) printing. (**a**) Microparticle bath serves as the embedding medium. (**b**) Bath chemistry triggers hydrogel crosslinking while particles support each layer. (**c**) Needle motion fluidizes particles locally, which then reassemble behind the nozzle. (**B**) 3D nanofiber-assisted embedding. Aligned PCL nanofibers (via divergent electrospinning) are printed first; cardiomyocytes are then deposited within the scaffold. (**C**) Microgel-based (MB) perfusable ventricle. (**a**,**b**) 3D design of a hierarchical vascular network. (**c**) Initial ventricle print using MB bioink in a Carbopol suspension. (**d**–**f**) Second print of a vascular network by extruding gelatin ink into the ventricle, which served as a temporary suspension medium. Scale bars: in (**c**–**f**) 5 mm. ((**A**) reproduced with permission from Shiwarski et al. [45]; (**B**) reproduced with permission from Wu et al. [39]; (**C**) reproduced with permission from Fang et al. [44]).

Naturally derived materials are highly compatible, support cell adhesion, and—because of their low viscosity, which generates less shear stress—often yield higher cell viability in extrusion-based bioprinting. As a result, they are frequently favored for cardiac tissue printing in research [39,40]. However, these materials often exhibit low mechanical stability (e.g., spreading) and have difficulty maintaining structural integrity after printing [39]. To enable continued use of these inks, researchers have developed embedding (or suspended) bioprinting, in which the construct is printed into a temporary support during extrusion to provide mechanical stabilization. A popular approach for cardiac applications is Freeform Reversible Embedding of Suspended Hydrogels (FRESH) [41]. In FRESH, the support bath—often composed of gelatin microparticles or yield-stress materials such as Carbopol—acts as a shear-thinning, self-healing medium that holds the extruded filament in place [42] (Figure 3A). Crosslinking agents are often included in the bath to assist bioink gelation. After printing, the support is gently removed thermally or chemically, leaving the 3D construct intact. This technique has proven highly promising for soft, low-viscosity bioinks while maintaining higher-resolution features [43].

Another embedding strategy for cardiac printing uses electrospun PCL nanofibers as a permanent support scaffold, as reported by Wu et al. [39]. Here, bioinks are extruded into the supporting nanofiber network, which remains attached to the construct after printing rather than being dissolved. The scaffold subsequently serves as a substrate for cell attachment, proliferation, and tissue maturation (Figure 3B). Beyond supporting low-viscosity prints, this approach provides topographical cues from the nanofiber architecture, promoting a high degree of cellular alignment.

A recent development in embedding bioprinting employed a microgel-based biphasic (MB) system, which functions both as a printable bioink and a suspension medium due to its shear-thinning and self-healing behavior [44]. Using this approach, the team printed a heart-shaped construct with open vessels via a sequential workflow (Figure 3C): (i) print the bulk geometry with the MB system in a temporary suspension state, (ii) directly write sacrificial inks into the still-soft MB structure to define perfusable vascular networks, and (iii) crosslink and remove the sacrificial phases. This process produced open-vessel channels within the bulk construct and enabled external continuous perfusion using a syringe pump.

While shear stress generated during extrusion-based printing can promote cardiac cell alignment, excessive shear from high extrusion pressure can cause cellular damage [46]. Knowing that shear stress depends on bioink properties, nozzle geometry, and applied pressure, Bonatti et al. [47] developed a deep-learning-based feedback system equipped with a camera that continuously monitors key extrusion parameters—pressure, nozzle speed, layer height, and infill density—and learns how each affects filament quality and shear generation [47]. When the model predicts shear approaching a cell-damaging threshold, it automatically slows the print, lowers the pressure, or switches to a larger nozzle to protect the cells. This adaptive approach can minimize shear stress while maintaining high print fidelity.

A recent innovation in embedding bioprinting further improves printing efficiency and parameter optimization. Sergis et al. [48] combined a high-resolution camera with real-time image-analysis software to evaluate how closely printed structures match the original 2D CAD. By automatically tracking features such as filament width, centerline drift, and excess material, the system quantified the performance of different bioinks and support baths within a single experiment—eliminating manual measurements. It can also reveal relationships among parameters; for example, the system could identify regions within a print that were prone to cell damage based on extrusion paths and layer geometry, thereby accelerating process optimization [48].

### 3.4. Volumetric Bioprinting

**Figure 4:**
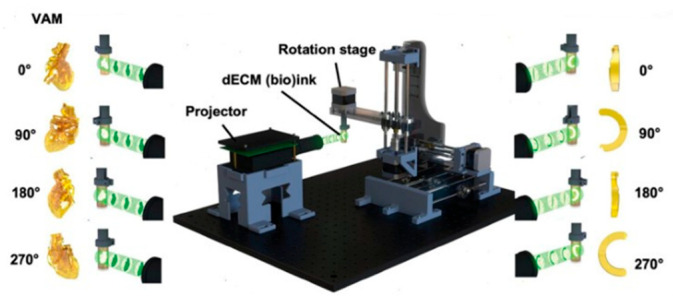
Schematics of volumetric bioprinting. (Figure reproduced with permission from Lian et al. [50]).

Volumetric bioprinting is an emerging light-based manufacturing technique that differs markedly from traditional layer-by-layer approaches. In this method, a cell-laden bioresin inside a rotating vial is exposed to a sequence of precisely controlled, spatially distributed light projections generated via computed tomographic reconstruction using a digital micromirror device (DMD) (Figure 4) [7]. These light patterns induce polymerization only within the voxels corresponding to the desired 3D structure, enabling the entire construct to form in a single, continuous step. This layerless process dramatically reduces fabrication time—often to seconds—and minimizes mechanical stresses on cells, preserving viability and delicate microarchitecture [49]. Because the build is not constrained by sequential deposition, it avoids issues such as interlayer delamination and prolonged light exposure that can harm embedded cells. Volumetric bioprinting also supports encapsulation at high cell densities. 

Volumetric bioprinting is particularly well-suited for producing entire organoids. In a study by Bernal et al. [49], liver epithelial organoids were volumetrically bioprinted within a specially tuned gelatin bioresin in under 20 s. The process preserved organoid morphology and polarity and achieved higher post-printing cell viability than traditional extrusion methods. Volumetric bioprinting has also been explored for cardiac tissue models. Lian et al. [50] formulated a heart-derived decellularized extracellular matrix (dECM) bioresin laden with cardiomyocytes and a photoinitiator system; solidification was triggered by visible light at 525 nm. The resulting cardiac constructs were printed in ~30–45 s, formed complex 3D geometries, expressed sarcomeric α-actinin, and exhibited synchronized contractions.

In another study, Jones et al. [51] replaced computed tomographic reconstruction with filamented light (Flight), a volumetric approach that projects multidirectional, coherent UV light into cell-laden GelMA to photopolymerize the construct within a 3D volume. They exploited modulation instability, which occurs as UV light interacts with the hydrogel during photopolymerization, to generate internal microfilament structures that provide physical cues for cardiomyocyte alignment. Printing was completed in <30 s, encapsulating high cell densities (~15–60 million cells/mL). Notably, they fabricated torsionally contracting cardiac structures that mimic native heart mechanics.

Volumetric bioprinting allows ultrafast fabrication, polymerizing entire 3D constructs within seconds, whereas DLP or extrusion-based systems typically require minutes to hours. This capability presents substantial potential for scalable production of organoids and heart-implantable constructs. Because no nozzle extrusion or layer deposition is involved, mechanical stress on cells is greatly reduced. Furthermore, the technique can simultaneously print multiple constructs without stage movement, offering an attractive solution for high-throughput applications such as drug screening and robotic tissue actuators.

Researchers have expressed concerns about resolution and spatial control, particularly when printing large constructs, since limited local heterogeneity may impede features such as vascular path formation. An innovative integration of volumetric and extrusion-based printing was explored by Ribezzi et al. [52] to create multimaterial, multicellular tissue models. Using a new “plug-and-play” support bath based on low–molecular-weight gelatin, fine extruded filaments were held in place and later incorporated into the final structure via volumetric crosslinking. Two wavelengths were used—one for alignment and one for curing—combining the speed and design freedom of volumetric printing with the precise material placement of extrusion, enabling the fabrication of detailed constructs such as functional cardiac organoids.

**Table 1:** Summary of bioprinting techniques characteristics.

Technique		Resolution [μm]	Bioink Viscosity Range [mPa·s]	Cell Concentration Range
Jetting-based [21]	Inkjet	~20–100	~3–10	~10^7^ cells/mL
	LIFT	~10–50	10–300	~10^8^ cells/mL
	EHD	<10	~1–100	~10^7^–10^8^ cells/mL
Stereolithography [29]		150–200	Not provided	~10^6^ cells/mL
DLP [32,33]		~30–50 (*XY*-axis)	Not provided	~5 × 10^6^ cells/mL
Extrusion-based		~250–300 [11,53]	100–10^5^ [5,53]	~10^6^ cells/mL [5,38]
	FRESH printing	~10–25 [43]	~10–100 [39,41]	~10^7^–10^8^ cells/mL [39,43]
Volumetric		~30–200 [50,51]	Not provided	~10^7^ cells/mL [51]

Different bioprinting techniques each offer unique advantages for cardiac tissue engineering, yet their practicality and maturity vary. Jetting-based methods such as inkjet, LIFT, and EHD bioprinting provide high precision and contactless deposition. It is well-suited to patterning complex microstructures such as Purkinje-like networks and heterogeneous cell densities. However, they remain limited by small construct thickness, narrow bioink viscosity ranges, and unstandardized parameters. Light-based approaches, including SLA and DLP, achieve excellent geometric fidelity, gradient crosslinking, and even 4D morphing to replicate myocardial curvature and anisotropy, though challenges with UV-induced cytotoxicity, especially when long curing times are required, and non-uniform cell distribution remain. Volumetric bioprinting stands out for its ultrafast, layerless fabrication that minimizes mechanical stress on cells and supports high cell densities, making it promising for producing large organoids or heart models, but its spatial resolution and limited material selectivity remain challenges.

Among all methods, extrusion-based bioprinting remains the predominant technique in the field due to its simplicity, versatility, and compatibility with a broad range of bioinks, especially soft, natural hydrogels native to the heart. It enables directional cell alignment through shear forces and shows robust development in real-time feedback control systems. With continued improvement in scalability, cytocompatibility, and structural control, the other modalities could be more broadly implemented for cardiac tissue fabrication, enabling faster and more efficient construction of complex, functional tissues. Continued advancements in visible-light photochemistry, hybrid volumetric–extrusion strategies and closed-loop process monitoring are expected to further enhance the efficiency and fidelity of these approaches in fabricating cardiac constructs.

## 4. Bioinks and Biomaterial Inks

Bioinks and biomaterial inks—natural or synthetic polymers and hydrogels designed to support living cells—should facilitate cell adhesion, proliferation, and differentiation [19]. Depending on the bioprinting method and application, bioinks (cell-laden) encapsulate cells during printing and can apply extensional stresses that promote cardiomyocyte alignment, particularly in extrusion-based bioprinting. Biomaterial inks (acellular), by contrast, are printed first to create a scaffold onto which cells are subsequently seeded—an approach that helps mitigate cytotoxicity concerns during printing, especially in light-based bioprinting [54].

### 4.1. Bioink Characteristics

The biophysical properties of bioinks and biomaterials play a critical role in tissue engineering because they directly influence how cells interact with one another and with their surrounding matrix [55]. For cardiac applications, bioinks must be carefully tuned to replicate the mechanical environment of the native myocardium while still supporting cell survival and function. Several rheological and structural parameters are commonly used to evaluate hydrogel performance in recent studies in cardiac applications, including stiffness (elastic modulus), viscoelastic behavior (G′ and G″), print fidelity, swelling ratio, and degradation resistance. Each of these parameters contributes to the delicate balance between structural support and biological compatibility.

The elastic modulus (or compressive Young’s modulus) describes material stiffness and is particularly important in cardiac tissues. If the bioink is too soft, it deforms easily and dissipates contractile forces rather than transmitting them between cardiomyocytes, which weakens coordinated and synchronous beating. Conversely, if the matrix is too stiff, it restricts cell spreading and migration, imposes mechanical load on cardiomyocytes and reduces their ability to shorten and contract effectively [8]. Hydrogel stiffness is also linked to a ‘mechanical memory’ effect, whereby cells “remember” their mechanical environment and alter their differentiation lineage accordingly; if the substrate is excessively stiff, this may activate fibrotic signaling. Therefore, the stiffness must be carefully tuned to support the desired long-term cell fate [55]. Complementing stiffness, most studies conduct the viscoelastic moduli (G′ and G″) measurements to assess how the material behaves as a solid (elastic response) versus a liquid (viscous response) [55]. For cardiac bioinks, post-crosslinking behavior should favor G′ > G″, indicating that the construct behaves more like a solid while retaining sufficient viscoelasticity for remodeling and mechanical flexibility.

Print fidelity, which is explicitly tested in extrusion-based bioprinting, ensures that the bioink maintains its intended geometry and resolution after printing; this depends on viscosity, shear-thinning behavior, and yield stress [55]. The swelling ratio determines how much water the hydrogel absorbs, affecting nutrient and oxygen transport as well as mechanical stability. Excessive swelling can weaken constructs or distort their shape, which becomes a significant parameter during implantation or in vivo testing [8]. Degradation resistance is needed so that the printed construct remains intact long enough to support cells through the maturation phase, before the cells themselves generate sufficient extracellular matrix [8]. These parameters can be tuned through polymer concentration, crosslinking chemistry and density, and the incorporation of additional molecules or secondary polymers. 

Bioink or biomaterial design represents a balancing act between printability, mechanical stability, and cell viability. For example, in extrusion bioprinting, higher viscosity improves print fidelity but increases shear stress during extrusion, which can damage cells. Low stiffness favors survival but risks structural collapse during culture [8]. In light-based bioprinting, fast or aggressive crosslinking enhances construct stability but may expose cells to toxic conditions [29,34]. For this reason, optimizing bioink properties is an iterative process, where the rheology must be adjusted to achieve both structural fidelity and biological support during tissue maturation.

### 4.2. Hybrid Hydrogel

Naturally derived biomaterials/bioinks offer high biocompatibility and bioactivity; for example, decellularized extracellular matrix (dECM) retains key growth factors, extracellular-matrix (ECM) proteins, and glycosaminoglycans (GAGs) [56]. However, these materials are typically mechanically soft due to the extraction process and the breakdown of native crosslinks; therefore, they tend to collapse after several printed layers, making printability unstable and difficult to control [57]. In contrast, synthetic biomaterials provide improved mechanical integrity, tighter control over printability, and consistent batch-to-batch properties. Yet, manufacturing processes—such as UV-induced or high-energy crosslinking—can generate cytotoxic by-products, often limiting direct cell encapsulation before printing [36]. To minimize this trade-off between biocompatibility and printability, recent studies have explored hybrid formulations and combinatorial strategies that enhance construct stability while supporting cell growth in CTE applications, including dual-crosslinking mechanisms, interpenetrating networks (IPNs), and rheological modulation.

In Shin et al. [56], Laponite-XLG nanoclay and polyethylene glycol diacrylate (PEG-DA) were combined with porcine-derived ventricular dECM to develop a mechanically tunable and printable dECM bioink. Laponite-XLG is a disk-like charged nanoclay that forms an ionic gel network, acting as a rheology modulator, increasing shear storage modulus and raising viscosity at rest, thereby improving filament stability after extrusion. PEG-DA enabled rapid photocrosslinking (<1 min) to lock the construct’s shape before sagging. By varying the concentration of PEG-DA, the authors achieved tunable stiffness values representing both healthy myocardium (≈13.4 kPa) and fibrotic myocardium (≈89 kPa), providing platforms for disease modeling and drug-screening applications [32].

Another study developed an IPN bioink comprising gelatin methacryloyl (GelMA) and methacrylated hyaluronic acid (MeHA) blended with dECM for cardiac tissue printing. GelMA provided cell-adhesive motifs and adjustable stiffness, whereas MeHA conferred slower degradation and greater rigidity [58]. A dual-crosslink method with UV light and microbial transglutaminase (mTGase) further enhanced stiffness and stability without excessive UV exposure. By varying GelMA/MeHA ratios, the team generated healthy and fibrotic myocardium analogs, showing that fibrotic tissues containing MeHA were ≈10× stiffer than those without, underscoring the influence of bioink composition on tissue mechanics [58].

Alginate remains a popular cardiac bioink due to its biocompatibility and instantaneous calcium-ion crosslinking capability [59,60]. However, similar to dECM and other naturally derived hydrogels, alginate-based systems exhibit low mechanical strength and poor shape retention. To improve these properties, recent formulations have integrated fibrinogen or secondary networks to enhance printability and post-printing stability. Budharaju et al. [5] incorporated fibrinogen into alginate to introduce cell-adhesion sites and generate a nanofibrous morphology after crosslinking, thereby better mimicking cardiac ECM. Fibrinogen also provided a secondary crosslinking mechanism through thrombin, which, together with calcium-mediated alginate gelation, enhanced construct stability. The study additionally pre-crosslinked alginate with low CaCl_2_, increasing viscosity, yield stress, and shape fidelity during extrusion. Several groups have further coupled embedding (support-bath) printing with calcium-ion additives to stabilize filaments as they are extruded [43]. However, one study suggested that such external crosslinking can lead to uneven gelation due to nonuniform calcium diffusion, potentially limiting oxygen/nutrient transport [59].

To address this limitation, Stola et al. [59] developed an internally crosslinked alginate dialdehyde (ADA)/alginate/gelatin bioink. By embedding insoluble CaCO_3_ and the pH-lowering agent glucono-δ-lactone (GDL) directly within the ink, gradual GDL hydrolysis releases protons that dissolve CaCO_3_, yielding a controlled, homogeneous Ca^2+^ release and triggering internal crosslinking. Gelation rate was tuned via CaCO_3_, GDL, and gelatin concentrations to achieve a usable printability window—stiffening late enough to permit extrusion but early enough to prevent sagging—eliminating the need for a support bath. Another recent report by Vettori et al. [8] added silk fibroin (SF) to alginate–gelatin bioinks and achieved hydrogel stiffness in the physiological range (≈38 kPa; native adult myocardium ≈10–50 kPa) with just 1% SF. SF introduced β-sheet and α-helix structures, providing additional intermolecular bonding that increased elasticity and enhanced contractile performance in the printed constructs.

### 4.3. Conductive Composite Hydrogel

Recent studies have incorporated conductive materials into bioinks for cardiac tissue printing to promote more synchronous and uniform cardiomyocyte contractions. Examples include carbon-based nanomaterials (graphene derivatives such as GO and rGO, and carbon nanotubes, CNTs), metallic particles (e.g., gold), MXenes, and conductive polymers such as poly(3,4-ethylenedioxythiophene) (PEDOT) [10,61]. The conductivities of these material-integrated cardiac constructs are summarized and compared with native adult myocardium in Table 2.

#### 4.3.1. Carbon-Based Nanomaterials

Graphene oxide (GO) is characterized by the presence of oxygen-containing functional groups on the surface of carbon atoms and is highly hydrophilic, supporting cell adhesion and spreading, but exhibits lower electrical conductivity because the oxygen functionalities disrupt electron pathways [61]. In contrast, reduced graphene oxide (rGO) contains fewer oxygen groups, resulting in higher conductivity, and has therefore been widely applied in cardiac tissue bioprinting. In Mousavi et al. (2024), rGO was incorporated into a GelMA-alginate methacrylate (AlgMA) bioink to bioprint ring-shaped cardiac tissues [53]. By controlling the degree of GO reduction—in this case using ascorbic acid (vitamin C) and tuning the reaction time and temperature—the authors were able to adjust stiffness, biocompatibility and conductivity. They achieved conductivity up to 1 × 10 ^−3^ S/cm with 0.2 mg/mL rGO, while the composite inks retained shear-thinning behavior, rapid structural recovery after extrusion (~4 s), and high printing fidelity. Tsui et al. [62] combined rGO with dECM and reported enhanced twitch force and accelerated contraction kinetics in printed cardiac constructs. The measured tissue conductivity ranged from 0.9 to 3.1 S/cm, significantly higher than that of native human myocardium, as it can promote electrical coupling and synchronization among immature cardiomyocytes.

Carbon nanotubes (CNTs) consist of graphene sheets rolled into hollow cylindrical structures with nanometer-scale diameters. They are attractive in tissue engineering, as they have large, negatively charged surface area that facilitates interactions with ionic species and adsorption of bioactive molecules [10]. Mehrotra et al. [36] developed a CNT-reinforced bioink composed of non-mulberry silk fibroin (nSF), GelMA and polyethylene glycol di-methacrylate (PEGDMA). GelMA and nSF are highly bioactive materials providing integrin-binding domains, while nSF additionally offers tensile strength comparable to native myocardium through β-sheet nanocrystal formation. The inclusion of CNTs within the 3D-printed construct was analogous to Purkinje fibers, suggesting that they may function as nanobridges to enhance electrical coupling and mechanotransduction. Indeed, CNT incorporation lowered electrical resistance, thereby increasing conductivity, and enhanced the stiffness of the printed construct compared to tissues without CNTs.

#### 4.3.2. Metallic Particles

MXenes are two-dimensional (2D) transition-metal carbides, nitrides, and carbonitrides with large surface areas and hydrophilic functional groups; their surfaces present many active sites that enhance adsorption of key ions [10]. In Basara et al. [63], soft poly(ethylene glycol) (PEG) hydrogels were first fabricated as supportive scaffolds, and Ti_3_C_2_Tx MXene patterns were then deposited directly onto the PEG surface via aerosol-jet printing. Because aerosol-jet printing achieves cell-scale resolution, it effectively guides single-cell alignment. Given PEG′s inherent resistance to non-specific cell adhesion, cardiomyocytes selectively attached and aligned along the MXene tracks. The study reported enhanced synchronous beating, expanded beating areas, and conduction velocities up to ~6.5 cm/s upon MXene integration.

A recent study compared CNTs and MXene nanosheets (Ti_3_C_2_) as conductive additives in alginate–gelatin hydrogels for cardiac bioprinting [64]. CNTs tended to increase viscosity and mechanical stiffness more readily, whereas MXenes altered viscosity less—potentially benefiting cell viability when printing with low-viscosity inks. A limitation observed for CNTs was aggregation at higher concentrations (tested ≥ 3 mg/mL), which reduced conductivity and compromised uniform network formation.

Another metallic nanomaterial used in cardiac bioprinting is gold nanostructures. In an early study, Zhu et al. [65] incorporated gold nanorods (GNRs) into a GelMA/alginate bioink for extrusion bioprinting. Gold enables stable biopolymer coatings—in this case, with GelMA—to minimize cytotoxicity while preserving conductivity. Experimentally, the printed constructs exhibited improved cell adhesion and retention, upregulated expression of connexin-43 and sarcomeric proteins, and suppressed excessive fibroblast proliferation, thereby helping maintain the cardiomyocyte–fibroblast balance crucial for maturation. Beyond enhancing conductivity, Ramirez et al. [66] demonstrated the use of gold as a contrast agent for non-destructive imaging of scaffold microarchitecture. They integrated PEG-coated gold nanoparticles (Au-NPs) into alginate–gelatin scaffolds and used micro-computed tomography (micro-CT) to visualize internal structures. Although conductivity was not directly evaluated, Au-NPs were uniformly dispersed, stable, and non-toxic to AC16 cardiomyocytes, maintaining >90% viability in 3D culture.

#### 4.3.3. Conductive Polymer

While several conductive polymers—such as polypyrrole (PPy), polythiophene (PTh), and polyaniline (PANI)—have been explored in tissue engineering, poly(3,4-ethylenedioxythiophene):poly(styrene sulfonate) (PEDOT:PSS), a PTh derivative, has emerged as particularly promising for cardiac tissue engineering. This is because PEDOT:PSS is an organic mixed ionic–electronic conductor, aligning with cardiac electrophysiology, in which ionic currents govern electrical signaling [67]. As a polymer, PEDOT:PSS is water-dispersible and forms stable, uniform networks, unlike nanoparticles that tend to aggregate. Testore et al. [67] demonstrated that integrating PEDOT:PSS into PEGDA/gelatin hydrogels allows tuning of the compressive modulus, swelling ratio, and degradation rate, and that PEDOT:PSS accelerates crosslinking kinetics—thereby reducing gelation time and improving construct stability [68]. More recently, Roshanbinfar et al. [68] developed an injectable collagen–PEDOT:PSS hydrogel for myocardial infarction therapy. Beyond faster gelation, PEDOT:PSS enhanced the hydrogel microarchitecture, yielding a structure more closely resembled native cardiac tissue. Encapsulated cardiomyocytes exhibited increased sarcomere length, stronger and faster contractions, and synchronized calcium transients.

**Table 2:** Summary of conductive biomaterials used in bioprinting cardiac tissues.

	Conductive Material	Other Composites in Bioink/Biomaterial	Conductivity [mS cm^−1^]	Cell Viability
Jalilinejad et al. [61]	Native adult myocardium	-	1.6 along; 0.05 across	-
Mousavi et al. [53]	Reduced graphene oxide	GelMA/AlgMA	1.0	>85% by day 7
Tsui et al. [62]		dECM	~0.9–3.1	>90% after 35 days
Mehrotra et al. [36]	Carbon nanotubes	nSF/PEGDMA/GelMA	Resistance ~93.6 kΩ (conductivity not reported)	Not provided
Basara et al. [63]	MXene	PEG	~1.1 × 10^7^	>90% by day 7
Zhu et al. [65]	Gold nanorods	GelMA	Resistance ~50 kΩ (conductivity not reported)	>70% after day 1
Ramirez et al. [66]	Gold nanoparticles	Alginate/gelatin	Not provided	>80% by day 2
Testore et al. [67]	PEDOT:PSS	PEGDA/gelatin	65.3 ± 4.8	>80% after day 1
Roshanbinfar et al. [68]	PEDOT:PSS	collagen	~0.00013 ± 0.00003	>85% at day 40

Although these results are promising for cardiac applications, reports on integrating PEDOT:PSS into bioprinted inks are still limited. Therefore, critical design parameters—including viscosity, printing-induced shear stress, and filament resolution—must be carefully optimized to ensure the material’s electroactive and mechanical advantages are preserved throughout the printing process.

All conductive additives integrated into biomaterials/bioinks have demonstrated enhanced conductivity compared with non-conductive controls, and most have also shown improved cardiac tissue functionality, particularly in contractile force and conduction velocity. However, the concentration of conductive additives must be carefully optimized and validated to ensure non-cytotoxic performance—for instance, 0.3 mg/mL rGO can reduce cell viability to 40% in Mousavi et al. [53]. The aggregation and percolation thresholds should likewise be identified by systematically modulating additive concentration, enabling the determination of a “sweet spot” where conductivity is maximized without compromising printability or biocompatibility [64].

Beyond concentration control, spatially patterned deposition of conductive fillers offers a strategy to minimize aggregation and prevent non-uniform stiffness within the printed constructs.

To complement the discussion above, the rheological and structural parameters of recent cardiac bioinks are consolidated in Table 3.

### 4.4. Shape-Morphing Bionks

4D bioinks—also called shape-morphing bioinks—enable printed constructs to undergo programmed structural changes over time in response to stimuli such as temperature, pH, magnetic fields, or electrical signals [31]. These transformations can include bending, folding, or reorientation, thereby mimicking the dynamic remodeling processes observed during cardiac development. This capability holds promise for replicating the native curved architecture of myocardium and for delivering constructs in compact forms that expand in situ to match patient-specific heart geometry, thereby improving fit, integration and functional performance [70].

Recent studies using 4D bioinks for cardiac patch fabrication highlight several design considerations compared to conventional bioinks [30,31].

First, printing modality is critical because certain techniques offer greater control over process parameters. For example, Miao et al. [30] used stereolithography and adjusted laser attenuation, intensity, and scanning speed to create crosslinking gradients. The resulting internal stress differentials drove 4D actuation, producing a curved cardiac patch [29,30].

Second, the stimulus-responsiveness mechanism must be carefully selected. Thermal triggers are among the most common: by defining a glass-transition temperature (Tg), the construct remains temporarily “fixed” below Tg and recovers its permanent shape above Tg—a phenomenon known as shape-memory behavior (SMP) [45]. For in vivo cardiac patches, Tg is typically tuned through crosslink density and network chemistry to operate near physiological temperature. Cui et al. [71] further demonstrated photothermal responsiveness by integrating graphene nanoplatelets within SMP matrices; near-infrared (NIR) light absorption generated localized heating, raising the temperature above Tg and triggering rapid shape recovery.

As 4D bioinks develop, several concerns have emerged. Biocompatibility and cytotoxicity must be carefully evaluated: although >60% cell survival was maintained after NIR-triggered shape recovery in one study, prolonged or high-intensity exposure may cause thermal damage [71]. Additionally, the constructs often exhibit MPa–GPa-scale stiffness—advantageous for maintaining programmed configurations but mechanically mismatched to the soft elasticity of native myocardium [31]. Because these patches are typically printed acellular and later post-seeded, achieving uniform and stable cell distribution before morphing is crucial. Finally, repeated folding/unfolding can introduce fatigue or microcracks; therefore, long-term mechanical durability under physiological loading must be thoroughly evaluated prior to clinical translation.

### 4.5. Bioinks in Volumetric Bioprinting

In volumetric bioprinting for cardiac tissue engineering, which relies primarily on light-based actuation, the bioink formulation must be carefully tuned to balance optical, chemical, and rheological properties.

First, optical transparency is critical: the material needs to allow sufficient and uniform penetration of visible or UV light throughout the rotating vial, as excessive absorbance or scattering from high solute concentrations can block light, limiting crosslinking depth and leading to poor resolution or incomplete structures [50]. Since volumetric printing enables very high cell densities, local variations in refractive index can also distort light paths; to address this problem, Jones et al. [51] added iodixanol to GelMA to match refractive indices throughout the vial and maintain uniform light propagation.

In parallel, the bioink must have adequate viscosity and stability during rotation to prevent sedimentation or diffusion that could blur the projected light patterns [50]. There is a trade-off between resolution and depth of crosslinking that needs to be carefully manipulated. As higher concentrations improve crosslink density and structural integrity, they also reduce light penetration, thereby lowering resolution [51].

## 5. Cell Types and Co-Culture Strategies

In cardiac tissue engineering, selecting appropriate cell types is crucial for fabricating constructs that faithfully replicate the native heart in both function and structure. Cardiomyocytes are employed in most studies to biomanufacture contractile cardiac tissues for either in vivo myocardial repair or in vitro drug-screening applications [72,73]. Moreover, co-culture systems have also emerged as an efficient strategy to more accurately recapitulate the cellular complexity and physiological interactions of human myocardium.

### 5.1. Cardiomyocytes

Adult human primary cardiomyocytes (hPCMs), directly isolated from adult hearts, are considered more physiologically relevant as in vitro models with high drug-response sensitivity. Wang et al. [74] reported that preparation—from cell extraction to bioink mixing—can be completed in about one day, making the process relatively efficient [74]. However, hPCMs are limited by their non-proliferative nature, short culture window, and pronounced donor-to-donor variability [73]. Consequently, most 3D-bioprinting studies employing primary cardiomyocytes have instead used cells derived from neonatal rats rather than adult human hPCMs [74,75], thereby introducing a developmental gap that alters ion-channel expression and contractile force generation and limits patient-specific drug screening fidelity.

To overcome these limitations, researchers have increasingly adopted stem cell-derived sources such as human embryonic stem cells (hESCs) and induced pluripotent stem cell-derived cardiomyocytes (iPSC-CMs), both possessing self-renewal and cardiomyogenic differentiation potential. iPSC-CMs, in particular, are favored for patient-specific applications and avoid the ethical concerns associated with hESCs [76]. Accordingly, iPSC-CMs have become the predominant cell source in cardiac tissue engineering [77]. Choosing between printing immature cells and promoting their maturation post-printing versus using more mature cardiomyocytes depends on the intended application, bioprinting constraints, and desired maturation approach. Immature cardiomyocytes resemble fetal CMs—they tolerate mechanical stress more effectively, proliferate readily, and exhibit higher engraftment potential—making them particularly suitable for implanted cardiac patches [77]. They also respond well to 3D-bioprinting cues—anisotropic patterning, tuned stiffness, printed vascular architecture and mechanical/electrical stimulations, which collectively promote structural and functional maturation, rendering 3D bioprinting a practical platform for tissues derived from immature cardiomyocytes [78].

By contrast, encapsulating more mature iPSC-CMs within 3D environments can yield stronger contractile function, improved structural organization, and reduced arrhythmogenic activity [79]. Although fewer studies have bioprinted fully matured cardiomyocytes, casting-based models have demonstrated clear advantages over very early-stage cells: enhanced sarcomere organization, partial isoform shifts toward adult contractile proteins (e.g., increased β-MHC, cTnI, and MLC2v), improved calcium-transient amplitude and kinetics, and more stable electrophysiology (e.g., more hyperpolarized resting potentials and reduced spontaneous activity) [78,80,81]. These traits translate to stronger, more coordinated contractions once embedded into engineered heart tissues or hydrogels, as extended culture time facilitates structural and electrophysiological maturation prior to 3D encapsulation [82].

For a practical timing window for iPSC-CM incorporation, it is suggested to wait until the onset of spontaneous beating in 2D culture (~day 10–14) to ensure basic electrophysiological competence for subsequent 3D cues [83]; ~2–4 weeks of pre-culture in 2D before embedding is often optimal, allowing cells to develop early sarcomeric organization, partial isoform switching, and improved Ca^2+^ handling, which together enhance subsequent 3D tissue performance [82].

Some strategies instead print pluripotent stem cells (iPSCs) directly and induce differentiation in situ, using geometrical confinement to guide maturation within 3D constructs. Ellis et al. [84] showed that encapsulating iPSCs in defined 3D geometries can direct tissue-level organization without external stimulation, improving myofibrillar alignment, Z-line formation, and anisotropic contractility. Similarly, Kupfer et al. [85] found that in situ dense cardiomyocyte networks (~100 million cells/cm^3^) and thicker muscle walls (~500 µm), approaching native myocardial structure. Because this strategy avoids cell dissociation or reseeding, which can disrupt gap junctions and sarcomeres, cells form intercellular junctions during differentiation, more closely mimicking in vivo development [85,86]. However, non-uniform cellularization and phenotypic heterogeneity remain major challenges: proliferating and differentiated cells may accumulate near the construct peripheries, leaving core regions sparsely populated, and mixed lineage outcomes may arise—beneficial for multicellularity and co-culture, yet reducing control over specification [85].

### 5.2. Co-Culture

Beyond single-cell-type approaches, co-culturing multiple cardiac cell populations has emerged as a powerful strategy to recapitulate the native heart’s complex multicellular architecture. While cardiomyocytes (CMs) remain central to electrical conduction and force generation, non-myocyte cells—particularly cardiac fibroblasts (FBs) and vascular endothelial cells (ECs)—play equally critical and complementary roles [87].

Cardiac fibroblasts (FBs) provide structural support by secreting extracellular matrix (ECM) proteins and facilitate deposition of cell-derived ECM as biomaterial scaffolds degrade. They are also key modulators of electrical cross-talk. Khoury et al. [88] explicitly examined heterocellular gap-junction coupling (HC) between FBs and CMs, showing that FB–CM electrical coupling can modulate impulse propagation and influence arrhythmogenesis. Elevated fibroblast density is a hallmark of aging and post-myocardial infraction (MI) remodeling, making FB-enriched co-cultures valuable for drug screening in fibrosis-associated arrhythmias and conduction disturbances.

Endothelial cells (ECs) are essential for promoting vascularization and facilitating nutrient and oxygen transport within engineered constructs [89]. Fabres et al. [90] used EC co-culture to recreate the in vivo CM–endothelium interface; under flow, ECs self-organized into a monolayer shell surrounding the tissue, which shielded CMs from shear stress and regulated drug diffusion kinetics, thereby better mimicking native tissue physiology. In cardiac patches, ECs can pre-form vascular networks that anastomose with host vasculature upon implantation, enhancing long-term perfusion and survival [91].

Other supporting lineages including mesenchymal stromal cells (MSCs) and smooth muscle cells (SMCs) are also studied and incorporated into the bioprinting process. MSCs contribute stromal and angiogenic support, recapitulating mesenchymal roles in vessel maturation [87]. In Brady et al. [91], MSCs promoted EC proliferation, and a subset differentiated into α-SMA^+^ pericyte-like cells that wrapped endothelial lumens, stabilizing and maturing microvessels. Smooth muscle cells (SMCs) serve as mural partners that stabilize EC organization and support capillary-like networks; Fabres et al. [90] reported that removing SMCs resulted in reduced EC coverage and compromised vessel integrity.

3D bioprinting offers unique advantages for co-culture systems, as it preserves multicellular organization (including spheroids) while defining precise geometries with high reproducibility [92]. It enables patterning strategies that control cell–cell proximity and spatial contact at micrometer scales—critical parameters for coordinated vascularization and regulated electrical conduction—thereby promoting hierarchical vessel formation over the disordered microvasculature typically seen in bulk hydrogels [35,91]. In aligned, micro-architected constructs, co-culture has been associated with enhanced synchronous contraction and lower excitation thresholds compared to CM-only tissues, reflecting improved electromechanical integration [90]. For example, Brady et al. [91] reported that patterned patches guided linear “trunk” vessels (~20–40 µm diameter) with branching microvessels (~5–15 µm), whereas non-patterned patches generated smaller, scattered vessels (<10 µm).

There are several key design considerations for establishing successful co-culture systems in 3D bioprinting, including medium compatibility, passage control, and cell-loading strategy. The culture medium must be optimized to support all cellular linages equally; for instance, researchers have employed a blended “Spheroid Medium” composed of a 2:1:1 ratio of CM-maintenance, endothelial, and fibroblast media, a 1:1 mixture of CM differentiation medium with EGM-2 for CM–EC co-cultures, or serum-free CM conditions supplemented with lineage-specific growth factors such as VEGF and bFGF to sustain non-myocytes [90]. Passage numbers—particularly for ECs and FBs—should be carefully controlled to minimize phenotypic drift and senescence-related changes that could impair function [88,93]. Finally, the sequence of cell deposition plays an important role: sequential or staged printing can outperform simultaneous loading by reducing competition for limited micro-niches and preserving cardiomyocyte alignment and functional coupling within the construct [92].

## 6. Applications

The preceding summaries of bioprinting modalities, biomaterials, and cell sources are synthesized into a decision framework that connects variable combinations to advances in 3D-bioprinting for cardiac tissue engineering—spanning cardiac patches, engineered tissues, organ-level constructs, and both disease-modeling and therapeutic applications.

### 6.1. Cardiac Patches

Cardiac patches have long served as temporary mechanical support systems following myocardial infarction (MI), aiming to restore cardiac function in damaged myocardium. However, several translational challenges remain—most notably insufficient vascularization, limited electrical and mechanical coupling with the host myocardium, incomplete innervation for physiological regulation, and only modest improvements in overall cardiac function and infarct size reduction after implantation.

Recent 3D bioprinting strategies have begun addressing these challenges by integrating advanced fabrication approaches to produce patches with geometrically optimized architectures, incorporating diverse cellular populations to promote vascular and stromal support, and functionalizing bioinks with conductive materials, bioactive molecules, or imaging and sensing components to enhance integration, monitoring, and therapeutic efficacy.

#### 6.1.1. Recent Bioprinting Techniques

Robust bioprinting techniques are increasingly being explored to overcome limitations of conventional cardiac patches, particularly their inability to remodel dynamically with the host heart or support long-term cardiomyocyte survival and vascularization [94].

In Cui et al. [29], beam-scanning stereolithography enabled fabrication of 4D, smart-material cardiac patches with self-morphing capacities and expandable microstructures. The printed patches adhered strongly to the epicardium in a murine chronic MI model in vivo, without the need for glues or sutures. The engineered wave-patterned fiber architecture promoted alignments of cardiomyocytes and endothelial cells, improving cell retention and capillary density, which collectively enhanced vascularization and reduced infract size. However, no significant improvement in cardiac function (as measured by ejection fraction) was observed, likely due to immature sarcomeric organization of the embedded cardiomyocytes. A mechanical mismatch in stiffness between the implanted patch and host myocardium was also noted, which impaired mechanical coupling and restricted synchronous deformation between the patch and native tissue.

**Figure 5:**
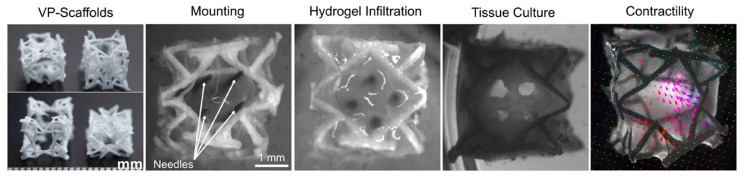
Biofabrication of reinforced cardiac tissues. Cardiac metamaterials are volumetrically printed, and are infiltrated with cardiomyocytes in a fibrin hydrogel and cultured, resulting in contractile cardiac tissues. White arrows indicate needles for supporting infiltration, purple arrows indicate tissue contraction direction. (Figure reproduced with permission from Jones et al. [94]).

In another study, Jones et al. [94] used volumetric 3D printing to fabricate anisotropic PCL metamaterials with myocardium-like stiffness and combined melt-electrowriting to produce fibrin-infiltrated meshes that filled inter-fiber gaps—effectively reducing permeability and enabling the patch to act as a hemostatic seal against wall rupture (Figure 5). The printed patch achieved directional Young’s moduli between 9 and 127 kPa, consistent with healthy myocardial values (20–100 kPa). This multimaterial demonstrated high suturability and reduced permeability and the ability to withstand ~80 mmHg intraventricular pressure in a porcine model, thereby restoring hemodynamic stability. However, chronic in vivo data, including vascularization, functional recovery and electrophysiology performance, remain necessary to fully evaluate translational potential.

#### 6.1.2. Cell Type

Recent cardiac patch studies have explored alternative and supportive cell types beyond cardiomyocytes, particularly those that drive myocardial repair through distinct biological mechanisms.

In one study, muscle-derived mesenchymal stromal cells (MSCs) were embedded within an extrusion-printed collagen patch; these cells primarily act through paracrine signaling to protect and preserve surviving myocardium, rather than through direct remuscularization [95]. Consistent with this mechanism, the printed MSC-laden patch exhibited strong angiogenic, anti-fibrotic, and anti-inflammatory activity in vitro. In vivo, left ventricular ejection factor (LVEF) improved by approximately 20% compared with the sham group, accompanied by an increase in stroke volume. However, infract size remained unchanged, aligning with the authors’ hypothesis that MSCs mainly promote myocardial preservation rather than tissue regeneration.

Another approach involved printing neural stem cells (NSCs) within a GelMA scaffold “pre-neuralized” using strontium-silicate microparticles. The released ions guided stem cell differentiation toward neuronal lineage, and the resulting patch enhanced innervation, leading to improved cardiomyocyte synchrony and maturation, increasing revascularization, and even modulation of circadian-related gene networks associated with cardioprotection and recovery [96].

Together, these studies highlight that the cell selection dictates the dominant repair pathway: stromal cells mainly provide immune-angiogenic support, while pre-neuralized neural cells deliver neural regulation that enhances electrical coordination and systemic healing responses.

#### 6.1.3. Bioinks Functionalization

In bioprinted cardiac patches, functionalizing bioinks with specialized biomaterials or biomolecules has become a common strategy to enhance conductivity, vascularization, and cell viability.

**Figure 6:**
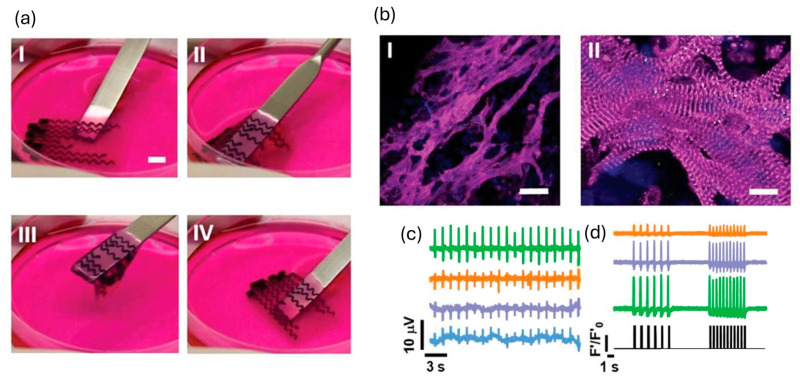
3D-printed stretchable micro-electrodes design for cardiac patches. (**a**) Flexible, soft patch; (**I**–**IV**) lifting and return to culture medium (scale bar, 6 mm). (**b**) Day-12 immunostaining for sarcomeric α-actinin (magenta) and nuclei (blue) (scale bars: (**I**), 50 µm; (**II**), 10 µm). (**c**) Simultaneous extracellular potential recordings from four sites. (**d**) On-patch electrical pacing (lower panel) with concurrent Ca^2+^ transients at three sites. (Figure reproduced with permission from Asulin et al. [97]).

To improve electrophysiological coupling, many studies have incorporated conductive additives—including conductive polymers, carbon nanotubes (CNTs), gold nanorods, and MXenes—which consistently lead to increased conduction velocity and synchronicity [63]. A notable advance by Asulin et al. [97] introduced a one-step, multi-material 3D-printing platform to fabricate a living cardiac patch with built-in soft electronics for pacing and sensing. The patch combined an ECM hydrogel loaded with cardiomyocytes, a graphite-in-PDMS conductive ink forming stretchable serpentine microelectrodes, and a dielectric PDMS passivation layer with exposed pads that established localized electrode–tissue interfaces (Figure 6). The hybrid construct showed excellent mechanical compliance, maintaining stable resistance after 1000 stretch/bend cycles, which is a performance compatible with myocardial strain. Functionally, it demonstrated bidirectional electrical capability, recording extracellular potentials and pacing tissues in vitro at 1–2 Hz using 7 V, 50 ms pulses. This integrated bioelectronic design holds potential for post-implant rhythm resynchronization and reduction in arrhythmic risk. However, the non-degradable and hydrophobic nature of the PDMS/graphite components raise concerns regarding long-term biofouling and foreign-body response, and in vivo durability and integration remain to be fully validated.

Biomolecule integration can further address post-MI inflammation and hypoxia to ensure the bioprinted cardiac patches maintain high cell survival before and after implantation. Mehrotra et al. [36] used bioprinting not only to fabricate an anisotropic, microfibrous scaffold reinforced with carbon nanotubes (CNTs) but also to incorporate boundary microcages for microinjection of calcium-peroxide (CPO)–GelMA and IL-10–GelMA microspheres. The CPO–GelMA component provided localized oxygen release to alleviate hypoxia, while IL-10–GelMA served as an immunomodulatory depot, polarizing macrophages toward a pro-healing M2 phenotype and enhancing patch adaptation. Bar et al. [98] incorporated macrophage-derived extracellular vesicles (EVs) loaded with miR-199a-3p into the bioink to counteract cell loss during printing, resulting in reduced apoptosis and improved early recovery outcomes.

Another study integrated CT-visible nanoparticles into the printed patch, opening opportunities for real-time, noninvasive imaging of implant fate and function [99]. By embedding gadolinium and gold nanoparticle contrast agents within the hydrogel bioink, the printed constructs became trackable under photon-counting CT, enabling quantitative assessment of structural integrity, spatial localization, degradation behavior, and even intravascular perfusion over time. This approach effectively transformed the patch into a theranostic platform, combining regenerative therapy with longitudinal imaging, thereby allowing clinicians to both repair injured myocardium and optimize treatment strategies through continuous visualization.

Despite these advances, current 3D-bioprinted cardiac patches still face several major challenges. Since most in vivo studies have been conducted in mice or other small animal models, patches remain limited in scale, and upscaling for clinically relevant dimensions will require re-optimization of parameters such as layer thickness, polymerization depth, and crosslinking kinetics. Furthermore, beating-frequency mismatches between human cardiomyocytes and host murine hearts can yield inconsistent contractile performance and non-translatable cardiac outcomes [29]. The duration of preclinical testing has also been relatively short—typically ~4 weeks—which may not sufficiently capture long-term remodeling, integration, or immune responses. In addition, arrhythmic risks may increase with enhanced electrical conductivity and higher cellular maturity, creating a trade-off that must be carefully balanced, especially when electronic components or conductive materials are incorporated [100]. Finally, the cell demand for clinically sized cardiac patches is enormous—on the order of hundreds of millions of cells per construct—posing significant manufacturing and scalability challenges for human trials [100]. Overcoming these limitations will be essential for advancing 3D-bioprinted cardiac patches toward safe and effective clinical translation.

### 6.2. Cardiac Tissues

3D-bioprinted cardiac tissues can serve as high-throughput drug-testing systems, preserving cell–cell and cell–matrix interactions as well as native-like architecture. To ensure these systems efficiently and robustly model pathological conditions or screening scenarios, the printed tissues must be sufficiently mature. Across the field, parameters used to enhance maturation in engineered heart tissues (EHTs) include refined structural patterning, vascularization, multimodal stimulation and prolonged 3D culture. An overview of recent bioprinted tissues and their maturation strategies is summarized in Table 4. Additionally, 3D bioprinting can directly recapitulate disease stages by incorporating pathological biomaterial cues into the printed constructs.

#### 6.2.1. Structural Patterning

Aligned cardiomyocytes can form well-organized sarcomeres and interconnect through gap junctions, as this is replicating the anisotropic property in the native heart. These are critical for synchronous contraction and proper electrical conduction, as well as leading towards a more mature state [17]. Three-dimensional bioprinting provides a powerful advantage by supplying precisely defined physical architecture that guides anisotropy from the moment cells are deposited or seeded.

Ahrens et al. developed a pre-alignment strategy using micropillar arrays loaded with collagen-based gels containing cardiomyocytes and fibroblasts [38]. Within these arrays, the cells self-assembled into elongated, contractile microtissues—termed anisotropic organ-building blocks (aOBBs)—that displayed highly aligned sarcomeres (Figure 2B). The harvested aOBBs were subsequently incorporated into bioinks and extruded into various tissue geometries, resulting in enhanced gap-junction formation and stronger adherens-junction connectivity. All quantitative and qualitative results are summarized in Table 4.

Similarly, Wu et al. [39] promoted tissue maturation through structural patterning by first fabricating highly aligned nanofiber scaffolds prior to cell incorporation. The printed PCL fibers, exhibiting alignment within ±10°, effectively guided cell elongation and orientation, producing more elongated nuclei and increased sarcomere length compared with flat substrates.

**Figure 7:**
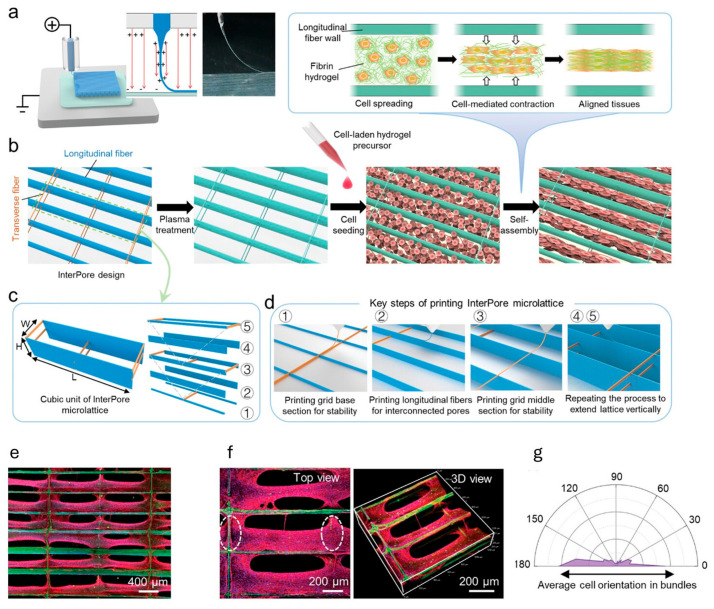
Patterned “InterPore” lattices fabricated by melt-electrohydrodynamic (EHD) printing. (**a**–**d**) Schematics of aligned, densely populated tissues within the InterPore microfibrous lattice: (**a**) melt-based EHD printing process; (**b**) concept of cell/hydrogel seeding and self-assembly into aligned bundles guided by the anisotropic lattice, white arrows indicate contraction directions; (**c**) cubic structural unit with transverse “V-shaped” fibers (L, H, W denote unit length, height, and width); (**d**) key printing steps. (**e**–**g**) Lattice characterization and guidance of cellular alignment: (**e**) F-actin–stained cellular bundles (red) embedded in a lattice containing green, fluorescent particles, showing bundle integrity and position; (**f**) top and 3D views of a magnified region from (**e**), with bundles traversing void gaps in the transverse fiber wall (highlighted in white circles); (**g**) histogram of nuclear alignment relative to the preferred orientation. (Figure reproduced with permission from Mao et al. [14]).

Integrating porosity is another patterning strategy that enhances cell–cell interconnectivity. Mao et al. [14] introduced “InterPore” lattices fabricated through melt-electrohydrodynamic printing, combining dense longitudinal fiber walls (≈400 µm spacing; ~50 layers) with staggered, V-shaped transverse triplets that created interconnected pores instead of isolated grid cells (Figure 7). This void-enriched architecture promoted fibrin remodeling, wherein cell traction forces compacted the fibrin and pulled encapsulated cardiomyocytes into highly aligned bundles traversing the transverse voids, preserving inter-pore connectivity. Compared to conventional lattices, these structures exhibited greater mechanical integrity and supported higher cell densities (~10^8^ cells/mL) [14].

As 3D bioprinting provides precise spatial control over bioink deposition, it enables the fabrication of constructs with diverse shapes and architectures. By varying printing patterns, structural designs, and the compositions or combinations of cells and biomaterials within a single construct, researchers can systematically investigate how these factors influence tissue behavior [38]. Moreover, 3D bioprinting allows the creation of suspended constructs, such as tissues anchored between micropillars, which not only promote cell compaction but also enable straightforward assessment of tissue contractility through force-measurement assays [101].

#### 6.2.2. Vascularization

Vascularization is a critical requirement for the maturation and long-term survival of 3D bioprinted cardiac tissues, as passive diffusion alone cannot support oxygen and nutrient delivery beyond ~200 μm, leading to hypoxia and necrosis in thick constructs [102]. A widely explored strategy in bioprinting is the co-encapsulation of endothelial cells (ECs) with cardiomyocytes and/or fibroblasts, allowing them to self-organize into vascular networks that integrate within the tissue, thereby promoting perfusion and supporting contractile function [11].

Lee et al. [41] developed a human-recombinant-tropoelastin (MeTro)/GelMA-based bioink to bioprint vascularized cardiac constructs. MeTro is an engineered elastin-like hydrogel precursor, provides stretchability, resilience and cell-binding motifs to support vascular cell attachment. This bioprinting strategy produced endothelial barrier with approximately twofold lower permeability than controls and cardiomyocytes exhibiting spontaneous beating with ~73% coordination by day 15 [41].

Incorporating gentle perfusion during culture further promotes barrier formation and vascular stability in bioprinted cardiac tissues. Fabres et al. [90] co-cultured hPSC-derived endothelial cells with smooth muscle cells, cardiac fibroblasts, and cardiomyocytes in a fibrin + Matrigel matrix, casting the tissues in a microfluidic chip and maintaining low-shear flow (via a rocking platform or 75 µL h^−1^ perfusion pump). Under these conditions, endothelial cells self-organized into a continuous monolayer encasing the cardiomyocyte core, forming a functional capillary-like barrier with reduced permeability and up-regulated endothelial-junction markers. Although demonstrated in a microfluidic format, the same principles are applicable to 3D bioprinting, where integrated perfusable channels and controlled low-shear flow can be used during maturation to enhance vascular integrity and tissue viability.

#### 6.2.3. Stimulations

Targeted stimulation is fundamental for maturing 3D engineered heart tissues (EHTs). In vivo, developing and adult myocardium experiences coordinated electrical excitation, cyclic mechanical loading, and sustained metabolic demand. In vitro, applying exogenous conditioning—such as electrical pacing, cyclic stretch, and dynamic/perfusion culture—recapitulates these cues and drives maturation. Recent EHT platforms therefore integrate one or more of these modalities to push tissues toward more adult-like structure and function.

**Figure 8:**
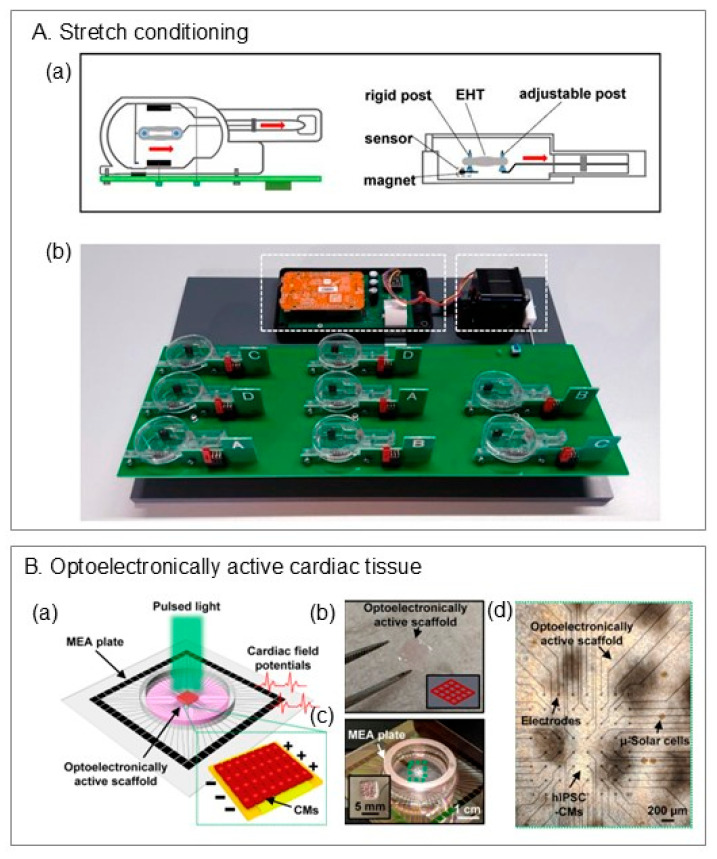
Recent mechanical and optical/electrical stimulations of 3D-bioprinted cardiac tissues. (**A**) Stretch-conditioning bioreactor. (**a**) Schematic of engineered heart tissue mechanically fixed in a biomimetic culture chamber; red arrow indicates stretch direction. (**b**) System setup: a microcontroller (left dashed box) acquires contraction data, generates pacing pulses, and drives BMCC platform rocking via a stepper motor (right dashed box). (**B**) Optoelectronically active cardiac tissue. (**a**) Schematic of light stimulation on a microelectrode array (MEA) plate. (**b**) Printed tissue (inset: CAD/model). (**c**) MEA plate with the optoelectronic scaffold. (**d**) Micrograph of cardiomyocyte-seeded scaffold. ((**A**) reproduced with permission from Lu et al. [12] (**B**) reproduced with permission from Ershad et al. [104]).

Lu et al. [12] used a custom biomimetic chamber providing progressive mechanical and electrical conditioning to accelerate EHT maturation [12]. Ring-mounted tissues were paced at 1 Hz and subjected to four daily stretch regimens over 3 weeks; the highest progression rate (0.32 mm day^−1^) yielded significant functional gains—twitch stress increased 5.1-fold relative to static culture, reaching ~11.3 mN mm^−2^ (Figure 8A). Tissues displayed physiological length-dependence (Frank–Starling behavior) and a positive force–frequency relationship, both hallmarks of functional maturation.

A complementary study compared four conditions—none, rocking only, electrical only, and combined electromechanical stimulation—to evaluate synergistic effects [103]. Electrical pacing used 30 V peak-to-peak, 4 ms pulses, stepped from 1 to 2.5 Hz; dynamic conditioning used 30 rpm rocking, inducing gentle flow and shear. The combined regimen yielded the most mature phenotype, showing enhanced sarcomere alignment and elongation, a force–frequency relationship sustained up to ~2 Hz, improved Ca^2+^ handling, and rocking-associated vascular-network formation when endothelial and mural cells were co-encapsulated [103].

To eliminate wired electrodes—and the contamination risk they introduce—Ershad et al. [104] developed an optoelectronically active scaffold that enables wireless pacing by light. They 3D-printed a GelMA scaffold embedding micrometer-scale silicon “μ-solar cells,” then seeded it with cardiomyocytes (Figure 8B). Under pulsed green light, the μ-cells generated distributed subthreshold photocurrents, modulating membrane potential and excitability; the construct was biocompatible and increased beat rate by ~20–44%.

Accordingly, these strategies are highly translatable to 3D-bioprinted cardiac tissues, where integrating electromechanical and optoelectronic cues can further promote synchronized contraction, enhanced calcium cycling, and functional maturation.

**Table 4:** An overview of recent bioprinted cardiac tissues in regenerative studies.

	Materials	Printing Method or Technique	Sarcomere Alignment	Sarcomere Length	Beat Rate	Contractile Force	Conduction Velocity	Expressions	Other Observations
Mature adult cardiomyocytes (CMs)	-	-	-	~2.2 µm [32]	~60–100 beats/min (bpm)	40–80 mN/mm^2^ [105]	~0.3–1 m/s [105]	↑ Sarcomere/contractile: *Myh6*, *Myl2*/*Myl3*, *Tnni3*, *Tnnt2*, *Ttn*; ↑ Calcium handling and coupling: *Ryr2*, *Casq2* (*Csq2*), *Atp2a2*/*SERCA2a*, *Pln*, *Gja1* (Connexin-43); ↑ Mito/FAO metabolism: *Fabp3*, *Cox6c*, *Uqcr11*, *Cpt1b*, *Cox8b* (adult isoform); ↑ *S100a2* ↓ immature contractile and glycolysis: *Myh7* (fetal myosin; mouse ventricle), *Pgam1*, *Ldha*, *Cox8a* (prenatal isoform); ↓ Proliferation/cell cycle: *Mki67*, *Ccna2*, *Ccnb1/2*, *Top2a*, *Prc1*; ↓ Transient CM–ECM (CME+) program: *Col1a1*/*Col3a1* [106,107]	
Ahrens et al. [38]	hiPSC-CMs, fibroblasts, collagen-based	Extrusion-based printing	~0.4 (1 = all orientation identical, 0 = orientations random)	sarcomere alignment measured by α-actinin positive cell area, not quantified	~0.8 Hz (=48 bpm, spontaneous)	~2 mN	~0.042 m/s	connexin 43 (CX43), N-cadherin, cTnT	-
Wu et al. [39]	hiPSC-CMs, PCL, GelMA	Electrospinning and extrusion-based printing	-	~2.2 µm	2 beats/230 s (=0.5 bpm, spontaneous)	-	-	Sarcomeric α-actinin, CX43	More elongated nuclei
Mao et al. [14]	hiPSC-CMs, ECs, PCL, fibrin	Melt-electrohydrodynamic	Clustered ~0° and 180°	~2 µm	~30 bpm (spontaneous)	-	~10–14 pixels/s	Sarcomeric α-actinin, *CX43*, *CD31*; sarcomeric markers: *MYOM2*, *MYL2*; excitation–contraction: *S100A1*, *CASQ2*, *GJA5*, *SCN5A*; metabolic: *CKMT2*, *PDK4*	More synchronized calcium waves, ~2.5 V/cm excitation thresholds
Lee et al. [41]	CMs, CF, ECs, MeTro/GelMA	Extrusion-based printing	-	-	~43 ± 3 bpm (spontaneous)	-	-	Sarcomeric α-actinin, CD31	Permeability coefficient of 0.8 × 10^−3^ cm/s;
Fabres et al. [90]	CMs, SMC, ECs, FBs, fibrin + Matrigel	microfluidics	-	-	~2.2 Hz (=132 bpm, spontaneous)	~0.12 mN	~1 mm/s	↑ Junction/EC barrier: *CDH5*, *CLDN5*, *TJP1/2*, *PTPRB*, *ESAM*; ↑ ECM remodel: *MMP2/9/16*, *COL1/3/12*, *ITGA5*; ↑ Connexins: *GJA1/5/4*; ↑ FA uptake/FAO: *LPL*–*GPIHBP1*–*CD36*, *CPT1B/2*, *ACADVL*, *ACAT1*; ↓ Glucose transport: GLUT2/4, *HK1*;	APD_90_ (stimulated at 2 Hz): ~180 ms; Permeability assay: Dextran-40 kDa FITC influx
Lu et al. [12]	hiPSC-CMs	Casting	-	2.19 ± 0.1 µm (under high stretch)	1 Hz (=60 bpm, after 1 week stimulation at 1 Hz)	~11.3 mN/mm^2^ (under high stretch)	-	↑ Adult phenotype: *MYH7*; ↑ excitation–contraction coupling, ↑ oxidative phosphorylation, and ↑ β-oxidation genes	Resting membrane potential: ~−72 mV; action potential (AP) amplitude: ~100 mV; AP upstroke velocity: ~13.5 V/s
Ershad et al. [104]	hiPSC-CMs	Extrusion-based printing	-	-	72 bpm (under light)	-	-	Sarcomeric α-actinin, connexin-43, cTnT	Cell viability >96% under light stimulation (no phototoxicity).

Note: ↑ indicates upregulation; ↓ indicates downregulation.

#### 6.2.4. Prolonged 3D Culture

Prolonged 3D culture fosters cardiomyocyte maturation by allowing time-dependent cell–cell and cell–matrix programs to develop within a stable, diffusion-permissive scaffold.

In Alonzo et al. [108], tri-culture cardiac organoids composed of cardiomyocytes, fibroblasts, and endothelial cells were encapsulated in 3D-printed gelatin–alginate lattices that provided long-term mechanical integrity and mass transport. The constructs were maintained for 21 days, during which progressive heterocellular coupling emerged. This was evidenced by an increase in connexin-43-positive junctions between cardiomyocytes and fibroblasts and CD31-positive endothelial networks that supported paracrine crosstalk. Enhanced cell viability and proliferation were observed with ECM deposition and mechanical stiffening, as cellularized scaffolds displayed higher storage and elastic moduli than acellular controls early in culture. The contractile activity of encapsulated cardiomyocytes was retained throughout the 21-day period, confirming stable excitation–contraction coupling in 3D.

Similarly, Maas et al. [109] fabricated cardiac spheroid systems encapsulating iPSC-CMs. In this model, cardiomyocytes self-aggregated within one day and underwent progressive organization over the following 2–3 weeks. By week 3, the spheroids displayed distinct α-actinin and troponin T striations. Transcriptomic analysis revealed upregulation of junctional (*GJA1*, *JPH2*, *PKP2*), desmosomal (*DES*), and mitochondrial (*ATP5A*) genes relative to extended 2D monolayers, consistent with improved electromechanical and metabolic competence. Functionally, calcium-handling parameters matured over this period: peak amplitudes increased by week 2, while rise/decay times and APD90 prolonged by week 3. However, cultures maintained beyond six weeks showed functional decline, indicating an optimal maturation window of two to three weeks.

#### 6.2.5. Disease Modeling

Lastly, 3D bioprinting not only enables the fabrication of tissue constructs with native-like cellular organization but also provides a powerful platform for disease modeling and drug screening.

Daly et al. [110] used bioprinting to create ring-shaped cardiac microtissues that model focal cardiac fibrosis by arranging spheroids into defined architectures. Healthy and fibrotic tissues were fabricated by varying the cardiomyocyte-to-fibroblast ratios (4:1 iPSC-CMs to CFs for “healthy”; 1:4 iPSC-CMs to CFs for “scarred”). A model of diseased myocardium was generated by printing rings composed of eight functional spheroids (representing healthy myocardial tissue) surrounding one fibrotic spheroid. These spheroids fused into cohesive tissues, showing robust cellular integration, sarcomere formation, and gap-junction development in healthy regions, whereas scarred areas exhibited increased fibroblast density and reduced electrical coupling. Diminished contractility and disrupted calcium signaling were also observed within scarred regions. Notably, treatment with miR302b/c enhanced cardiomyocyte proliferation and restored both contractile and electrophysiological function, demonstrating the model’s capability to evaluate therapeutic interventions [110].

**Figure 9:**
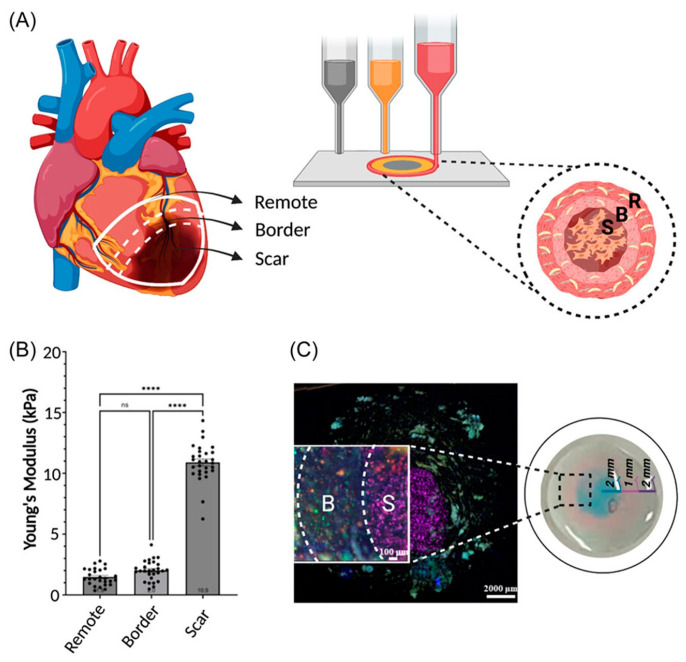
3D bioprinted post–myocardial infarction (MI) tissue model. (**A**) Schematic of three regions: remote (R), border (B), and scar (S). (**B**) Young’s modulus of composite hydrogels representing each region. (**C**) Tiled fluorescence image of the printed model: hiPSC-CMs (CellTracker Green), hiPSC-CFs (CellTracker Blue), hiPSC-ECs (CellTracker Orange), and hiPSC-CMFs (CellTracker Deep Red). Inset: magnified view (n = 3, ns: nonsignificant, **** *p* < 0.0001). (Figure reproduced with permission from Basara et al. [111]).

Another study [111] engineered an aged human post-MI myocardium using a three-printhead extrusion-based 3D bioprinter to fabricate three concentric regions—remote, border, and scar (Figure 9). Each region was printed with region-specific bioinks (remote: 10% GelMA/1 mg mL^−1^ collagen/0.025% PI; border: 12% GelMA/1.5 mg mL^−1^ collagen/0.033% PI; scar: 15% GelMA + 1% MeHA/2 mg mL^−1^ collagen/0.15% PI) and distinct aged cell compositions (remote: hiPSC-CMs/hiPSC-CFs/hiPSC-ECs at 20/6/3 M mL^−1^; border: the same plus hiPSC-myofibroblasts (CMFs) at 10/3/1.5/1; scar: hiPSC-CMFs only at 10). Mechanical testing confirmed region-specific stiffness gradients, while immunostaining verified appropriate cell phenotypes. The resulting construct provided a functional aged post-MI model that can serve as a testbed for studying fibrosis progression and evaluating regenerative therapies.

In a different approach to cardiac disease modeling, Miller et al. [112] used 3D bioprinting to assess the toxicity of copper oxide nanoparticles (CuO NPs) on human iPSC-derived cardiac microtissues. The model provided a means to study toxicological stress in 3D cardiac constructs, revealing dose-dependent impairments in tissue function and identifying mitochondrial damage and apoptosis activation as primary mechanisms of CuO NP toxicity, with a lethal dose (LD50) of 7.176 μg/mL. Moreover, the platform demonstrated how 3D-bioprinted cardiac tissues can be used to assess the impact of toxic agents and evaluate potential therapeutic interventions that might mitigate the damage, paving the way for novel treatments for environmentally induced cardiovascular diseases.

### 6.3. Cardiac Organoids

3D bioprinting is especially valuable for biomanufacturing irregular, large, and complex geometries such as the heart. As research progresses from small-scale cardiac tissues to organ-scale, chambered constructs, investigators are leveraging innovations including embedded printing for continuous builds, modular assembly, and integrated perfusion-flow conditioning to achieve target shape and function.

Fang et al. [44] introduced a novel sequential embedded bioprinting approach (SPIRIT) to print a ventricular model with open, perfusable blood vessels. They used a microgel-based biphasic (MB) bioink, capable of serving both as a printable bioink and a suspension medium, providing structural support while allowing subsequent printing of secondary inks. In the first stage, the external ventricle geometry was printed within a support bath. Without crosslinking the construct, they then performed a second embedded printing step in which sacrificial endothelial cell (HUVEC)-laden gelatin ink was written directly inside the freshly printed ventricle to generate freeform vascular channels. Upon removal of the sacrificial ink, these channels were perfusable and became uniformly lined with endothelial cells, forming a confluent endothelium by day 7 and even showing endothelial invasion into the surrounding matrix. The printed ventricle constructs achieved high shape fidelity (>95% match to CAD), hierarchical vascular trees, and—when loaded with cardiomyocytes—enhanced viability, spontaneous calcium transients, and sarcomeric α-actinin and connexin-43 expression, confirming preliminary functional maturation.

**Figure 10:**
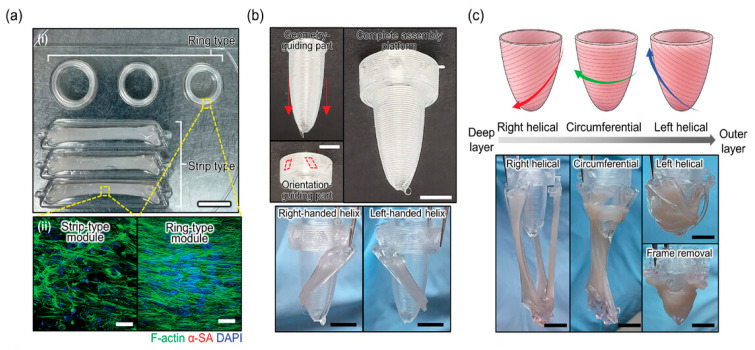
Reproducing myocardial fiber orientation in a chamber-like construct and left-ventricular twist. (**a**) EHT modules of varied shapes/sizes used to program myocardial fiber orientation (MoCha assembly): (**i**) gross view (scale bar, 1 cm); (**ii**) immunofluorescence—F-actin (green), α-sarcomeric actin (red), DAPI (blue) (scale bar, 50 µm). (**b**) Assembly platform with orientation- and geometry-guiding fixtures and the resulting construct, with red arrows indicating the direction of the tilted channels, and titled holes (circled in red) enabling EHT modules to form right- and left-handed helical orientations; images show right- and left-handed helices formed from a single module (scale bars, 5 mm). (**c**) Schematic of layer-dependent fiber orientations and images of the MoCha assembly process (scale bars, 5 mm). Figure reproduced with permission from Hwang et al. [113]).

A recent innovation seeks to recreate the myocardial fiber orientation, as native left ventricular function depends on transmural fiber helices that generate left-ventricular twist (LVT) during contraction and relaxation [113]. A recent study used a bioprinting-assisted tissue assembly (BATA) approach to build a modular ventricle. It first printed uniaxially aligned EHT modules containing iPSC-derived cardiomyocytes and cardiac fibroblasts in hdECM. These were printed onto PEVA post frames to induce tension, compaction, and alignment. These modules were then assembled like “LEGO” blocks to form multi-layer structures with programmable fiber directions (Figure 10). Individual modules showed spontaneous and paced contraction, optical mapping signals, and drug responses (isoproterenol and omecamtiv increased inotropy; nifedipine decreased it). After assembly, modules fused structurally through fibroblast-mediated ECM remodeling and formed connexin-43 junctions without losing structure. The final “MoCha” chamber exhibited synchronized beating, calcium wave velocity of ~1.4 cm/s, and a net left-ventricular twist of ~0.68°. Although this is lower than the native 12–15°, it demonstrates successful fiber programming toward anisotropic contraction [114].

Beyond the single-chamber construct, researchers are now creating multi-chamber heart models with perfusable geometries. Kupfer et al. [85] developed a 3D bioprinted, chambered human cardiac muscle pump (hChaMP) by printing hiPSCs in an ECM-based bioink and differentiating in situ. The constructs formed muscular walls of 100–500 µm, with ~88% cardiomyocytes, and exhibited synchronous beating at ~40–80 BPM for at least 6 weeks. They generated pressure oscillations of ~0.3–0.5 mmHg, pressure–volume loops, and stroke work of ~14.5 nJ with a maximum ejection fraction of 6.5%. The constructs also exhibited robust electrical coupling and dose-dependent pharmacological responses.

Zeng et al. [114] further expanded this concept by designing two- and four-chamber heart models using DLP printing with a conductive PGI hydrogel ink. They embedded helical micro-grooves in ventricular walls and circumferential grooves in atrial walls and integrated a hollow auxetic re-entrant layer to reproduce anisotropic stiffness. They further included vessel inlets and tri-leaflet valves to achieve unidirectional flow. After NaOH strengthening and ECM coating, the scaffolds were seeded with hiPSC-derived cardiomyocytes, which aligned along the patterned grooves, formed anisotropic tissues, and established gap junction coupling. The two-chamber models achieved valve-mediated one-way pumping, while the four-chamber model displayed synchronized contractions with ventricular-like torsional twist (~7°), sustained over 10 million contraction cycles without structural failure. These results demonstrate that structurally encoded scaffolds can guide organ-level organization and function.

Despite rapid progress toward organ-level constructs, several limitations persist. Viable tissue thickness remains below native myocardium, and uniform cell infiltration is challenging, with viability often concentrated at the periphery [85]. Immaturity of cardiomyocytes further limits function [114]. Ventricular twist angles also fall short of physiological ranges—likely due in part to coarse, layer-by-layer fiber orientations; recent work suggests that imposing a gradual transmural rotation in fiber angle, rather than abrupt changes between layers, can better recapitulate native mechanics [113,114]. Even so, it is noteworthy that complex, chambered geometries produced by 3D additive manufacturing can remain structurally stable in vitro for over a month.

## 7. Limitations and Future Directions

Despite major advances, fully adult-like phenotypes remain difficult to achieve in bioprinted cardiac tissues. Many constructs still fall short on twitch stress, show only modest conduction velocities, and lack consistent positive force-frequency relationships [12,38,103,104]. Throughput is another bottleneck: few groups have scaled 3D bioprinting for high-content drug screening. As tissues are miniaturized and produced in large batches, maturity often declines, and studies on compatible platforms that combine high-throughput printing with standardized stimulation (electrical, mechanical, and flow) are limited [4]. The cell numbers required for organ-scale builds, which involve billions of cardiomyocytes derived from hiPSCs along with fibroblasts, endothelial cells, and conduction cells, remain costly and labor intensive [115]. Equally critical is the need for bioinks that support very high cell densities without excessive shear damage during extrusion while still delivering the biochemical and mechanical cues needed for survival and self-organization [115]. Variability across hiPSC lines further contributes to inconsistent tissue formation and maturation [4].

A practical path forward is emerging: closed stirred tank bioreactors that expand hiPSC aggregates to the multibillion-cell scale within days, followed by direct incorporation into high-density bioinks for printing and differentiation after printing [116]. Pairing these tissues with perfusion electromechanical bioreactors, which combine controlled flow, progressive mechanical load, and paced stimulation, should push constructs toward adult-like force and conduction [117]. In parallel, closed-loop printing with in-line quality control can limit wall shear and stabilize fidelity by integrating camera, force, and impedance feedback during extrusion, with optical mapping after printing to quantify synchrony [47,48]. Finally, combining machine learning surrogates with multiphysics models can reduce trial and error by predicting how fiber angle gradients and vascular topology influence performance; comparing these digital predictions with measured readouts will accelerate iteration and standardize quality assessment [113,114].

## 8. Conclusions

3D bioprinting is reshaping cardiac tissue engineering by uniting precise architecture, increasingly tunable bioinks, and rational cell sourcing to produce constructs that begin to recapitulate myocardial structure and function. Jetting, light-based, extrusion, and volumetric approaches each contribute unique strengths in resolution, speed, and cell handling, while hybrid natural–synthetic bioinks, conductive composites, and shape-morphing formulations provide biochemical, mechanical, and electrical cues that steer alignment, coupling, and maturation. Co-culture strategies with fibroblasts, endothelial cells, smooth muscle cells, and conduction-relevant populations further stabilize function and enable vascularization, and emerging chambered constructs demonstrate that organ-scale geometry can be paired with measurable pump-like behavior. At the same time, consistent adult-like phenotypes remain challenging, thick viable tissues require robust perfusion, and scalable manufacturing with in-line quality control is still developing. Overall, the field is working toward more reproducible in vitro platforms for drug discovery and disease modeling, and continued advances are expected to move engineered constructs from proof of concept toward reliable tools for science and therapy.

## Figures and Tables

**Table 3:** An overview of bioinks design parameters from recent bioprinted cardiac tissues in regenerative studies.

	Bioink Composition	Bioprinting Technique	Young’s Modulus (Compressive)	Shear Elastic Modulus (G′) Post-Crosslink	Shear Viscous Modulus (G″) Post-Crosslink	Print Fidelity	Swelling Ratio	Degradation Resistance	Physiological Results
Native tissues	-	-	~10–50 kPa [8]	~2.5–2.9 kPa (porcine)	~0.6–0.8 kPa (porcine) [69]	-	-	-	-
Shin et al. [56]	dECM/Laponite-XLG/PEG-DA	extrusion	~13.4 ± 0.4 kPa (healthy myocardium)	~0.761 kPa	Not provided	~2.86 (filament width/nozzle ID)	~0 (after 7 days)	Not provided	>94% cell viability after 7 days; Beating velocity: 8.0 ± 2 µm/s; Beating frequency: 0.65 ± 0.26 Hz; Better sarcomeric α-actinin striation and connexin 43 expression with dhECM.
Basara et al. [58]	dECM/MeHA/GelMA	extrusion	~2.8 kPa (healthy myocardium)	~4.7 kPa	~2.3 kPa	~0.8 (line spacing target/measured)	12 ± 3% after 24 h	~degraded in 5 h (enzymatic digestion with collagenase)	Beating velocity: 8.0 ± 2 µm/s; Beating frequency: 0.65 ± 0.26 Hz; Better sarcomeric α-actinin striation and connexin 43 expression with dECM.
Stola et al. [59]	ADA/alginate/gelatin	extrusion	~2–6.8 kPa	~0.65–1.3 kPa	Not provided	~1.3–2.5 (filament width/nozzle ID)	~7–21% increase in weight over 21 days (for 0–20% gelatin content)	~30–80% weight loss after 24 h (dependent on gelatin concentration)	cell viability: >50%; cell adhesion observed in 25% gelatin content
Budharaju et al. [5]	Alginate/fibrin	Cardiac tissue	64.81 ± 21.89 kPa	~123 ± 10 kPa @100 rads	Not provided	2.33 ± 0.2 (filament width/nozzle ID)	~1783 ± 187% after 6 h	48.0 ± 4.1% mass loss at day 14	cell viability: >80%; high proliferation observed; shown sarcomeric α-actinin and connexin-43 cardiac marker expression
Vettori et al. [8]	Silk fibroin/alginate/gelatin	extrusion	38 kPa	~8 kPa	~0.7 kPa	~0.6 (filament width/nozzle ID)	Not reported	~91% intact at day 28	cell viability: ~2 toxicity ratio; immunostaining confirmed CD31+, vimentin+, and troponin T+ cells; ~0.02 Hz contraction frequency
Mousavi et al. [53]	rGO/GelMA/AlgMA	extrusion	29.9 ± 2.6 kPa	~50 kPa @0.1–10% rad/s	~10 kPa @0.1–10% strain	Not provided	~800% after 24 h (mass gain relative to dry mass)	59% mass loss in 28 days	cell viability: >85%; confirmed cTnT, α-actinin, connexin-43, and F-actin staining in cells; confirmed spontaneous beating in cells at ~36 ± 5 BPM by day 7
Tsui et al. [62]	rGO/dECM	extrusion	17.5 ± 0.5 kPa	~0.5 kPa @0.1–100% rad/s	~0.12 kPa @0.1–100% rad/s	Not provided	Not provided	Not provided	Twitch force: ~45 µN by day 28–35; sarcomere length up to 2.11 µm; Conduction velocity: 35.4 ± 2.3 cm/s at Day 35
Mehrotra et al. [36]	CNTs/nSF/PEGDMA/GelMA	extrusion	51.4 ± 4.5 kPa	~0.05 kPa @0.01–1% rad/s	~0.005 kPa @0.01–1 rad/s	~0.88 (filament width/nozzle ID)	80%	Not provided	Gene expression upregulated; Beating rate: 102 ± 5 bpm (CNT) vs. 76 ± 6 bpm (no CNT); Sarcomere z-lines: 1.5–2.5 µm
Basara et al. [63]	MXene (Ti_3_C_2_T*_x_*)/PEG	Aerosol jet printing	17.5 ± 5.5 kPa	Not provided	Not provided	Not provided	Not provided	Not provided	cell viability: 93%; gene expressed: *MYH7*, *TNNT2*, SERCA2 and connexin 43; conduction velocity: 6.5 cm/s
Zhu et al. [65]	GNRs/GelMA	Extrusion	~4.2–4.7 ± 0.3 kPa	Not provided	Not provided	Not provided	Not provided	~100% intact at day 5 (shown by no GNRs leaking)	gene expressed: Cx-43 and troponin I; synchronous beating from day 2; enhanced cardiomyocytes adhesion
Ramirez et al. [66]	Au-NPs/alginate/gelatin	Extrusion	~18.5 kPa	6.15 ± 0.19 kPa	0.32 ± 0.08 kPa	Not provided	Not provided	Not provided, but biodegradation observed	cell viability: >90%

## Data Availability

No new data were created or analyzed in this study. Data sharing is not applicable to this article.

## References

[1] Shadrin I.Y., Allen B.W., Qian Y., Jackman C.P., Carlson A.L., Juhas M.E., Bursac N. (2017). Cardiopatch Platform Enables Maturation and Scale-up of Human Pluripotent Stem Cell-Derived Engineered Heart Tissues. Nat. Commun..

[2] Agarwal T., Fortunato G.M., Hann S.Y., Ayan B., Vajanthri K.Y., Presutti D., Cui H., Chan A.H.P., Costantini M., Onesto V. (2021). Recent Advances in Bioprinting Technologies for Engineering Cardiac Tissue. Mater. Sci. Eng. C.

[3] Heinrich M.A., Liu W., Jimenez A., Yang J., Akpek A., Liu X., Pi Q., Mu X., Hu N., Schiffelers R.M. (2019). 3D Bioprinting: From Benches to Translational Applications. Small.

[4] Cho S., Discher D.E., Leong K.W., Vunjak-Novakovic G., Wu J.C. (2022). Challenges and Opportunities for the next Generation of Cardiovascular Tissue Engineering. Nat. Methods.

[5] Budharaju H., Sundaramurthi D., Sethuraman S. (2023). Efficient Dual Crosslinking of Protein–in–Polysaccharide Bioink for Biofabrication of Cardiac Tissue Constructs. Biomater. Adv..

[6] Ainsworth M.J., Chirico N., de Ruijter M., Hrynevich A., Dokter I., Sluijter J.P.G., Malda J., van Mil A., Castilho M. (2023). Convergence of Melt Electrowriting and Extrusion-Based Bioprinting for Vascular Patterning of a Myocardial Construct. Biofabrication.

[7] Nuñez Bernal P., Delrot P., Loterie D., Li Y., Malda J., Moser C., Levato R., Bernal P.N., Li Y., Malda J. (2019). Volumetric Bioprinting of Complex Living-Tissue Constructs within Seconds. Adv. Mater..

[8] Vettori L., Tran H.A., Mahmodi H., Filipe E.C., Wyllie K., Liu Chung Ming C., Cox T.R., Tipper J., Kabakova I.V., Rnjak-Kovacina J. (2024). Silk Fibroin Increases the Elasticity of Alginate-Gelatin Hydrogels and Regulates Cardiac Cell Contractile Function in Cardiac Bioinks. Biofabrication.

[9] Park S.H., Park J.Y., Ji Y.B., Ju H.J., Min B.H., Kim M.S. (2020). An Injectable Click-Crosslinked Hyaluronic Acid Hydrogel Modified with a BMP-2 Mimetic Peptide as a Bone Tissue Engineering Scaffold. Acta Biomater..

[10] Elkhoury K., Patel D., Gupta N., Vijayavenkataraman S. (2025). Nanocomposite GelMA Bioinks: Toward Next-Generation Multifunctional 3D-Bioprinted Platforms. Small.

[11] Bera A.K., Rizvi M.S., Kn V., Pati F. (2024). Engineering Anisotropic Tissue Analogues: Harnessing Synergistic Potential of Extrusion-Based Bioprinting and Extracellular Matrix-Based Bioink. Biofabrication.

[12] Lu K., Seidel T., Cao-Ehlker X., Dorn T., Batcha A.M.N., Schneider C.M., Semmler M., Volk T., Moretti A., Dendorfer A. (2021). Progressive Stretch Enhances Growth and Maturation of 3D Stem-Cell-Derived Myocardium. Theranostics.

[13] O’Connell C., Ren J., Pope L., Li Y., Mohandas A., Blanchard R., Duchi S., Onofrillo C. (2020). Characterizing Bioinks for Extrusion Bioprinting: Printability and Rheology. Methods Mol. Biol..

[14] Mao M., Han K., Gao J., Ren Z., Zhang Y., He J., Li D. (2025). Engineering Highly Aligned and Densely Populated Cardiac Muscle Bundles via Fibrin Remodeling in 3D-Printed Anisotropic Microfibrous Lattices. Adv. Mater..

[15] Liu N., Ye X., Yao B., Zhao M., Wu P., Liu G., Zhuang D., Jiang H., Chen X., He Y. (2021). Advances in 3D Bioprinting Technology for Cardiac Tissue Engineering and Regeneration. Bioact. Mater..

[16] Derakhshanfar S., Mbeleck R., Xu K., Zhang X., Zhong W., Xing M. (2018). 3D Bioprinting for Biomedical Devices and Tissue Engineering: A Review of Recent Trends and Advances. Bioact. Mater..

[17] Ligon S.C., Liska R., Stampfl J., Gurr M., Mülhaupt R. (2017). Polymers for 3D Printing and Customized Additive Manufacturing. Chem. Rev..

[18] Deo K.A., Singh K.A., Peak C.W., Alge D.L., Gaharwar A.K. (2020). Bioprinting 101: Design, Fabrication, and Evaluation of Cell-Laden 3D Bioprinted Scaffolds. Tissue Eng. Part A.

[19] Wu C.A., Zhu Y., Woo Y.J. (2023). Advances in 3D Bioprinting: Techniques, Applications, and Future Directions for Cardiac Tissue Engineering. Bioengineering.

[20] Zhang T., Wan L.Q., Xiong Z., Marsano A., Maidhof R., Park M., Yan Y., Vunjak-Novakovic G. (2012). Channelled Scaffolds for Engineering Myocardium with Mechanical Stimulation. J. Tissue Eng. Regen. Med..

[21] Ng W.L., Shkolnikov V. (2024). Jetting-Based Bioprinting: Process, Dispense Physics, and Applications. Biodes. Manuf..

[22] Pu X., Wu Y., Liu J., Wu B. (2024). 3D Bioprinting of Microbial-Based Living Materials for Advanced Energy and Environmental Applications. Chem. Bio Eng..

[23] Xu T., Zhao W., Zhu J.M., Albanna M.Z., Yoo J.J., Atala A. (2013). Complex Heterogeneous Tissue Constructs Containing Multiple Cell Types Prepared by Inkjet Printing Technology. Biomaterials.

[24] Zhu H., Li R., Li S., Guo K., Ji C., Gao F., Zheng Y., Zhu R., Wang H., Zhang L. (2024). Multi-Physical Field Control Piezoelectric Inkjet Bioprinting for 3D Tissue-like Structure Manufacturing. Int. J. Bioprinting.

[25] Yang Z., Tian H., Wang C., Li X., Chen X., Chen X., Shao J. (2022). Actuation Waveform Optimization via Multi-Pulse Crosstalk Modulation for Stable Ultra-High Frequency Piezoelectric Drop-on-Demand Printing. Addit. Manuf..

[26] Koch L., Deiwick A., Soriano J., Chichkov B. (2023). Laser Bioprinting of Human IPSC-Derived Neural Stem Cells and Neurons: Effect on Cell Survival, Multipotency, Differentiation, and Neuronal Activity. Int. J. Bioprinting.

[27] Ji P., Heinle J.S., Birla R.K. (2024). Development of a Novel Method to Fabricate Highly Functional Human Purkinje Networks. bioRxiv.

[28] Cho Y.H., Kang J.W., Choi S.H., Yang D.H., Anh T.T.X., Shin E.S., Kim Y.H. (2019). Reference Parameters for Left Ventricular Wall Thickness, Thickening, and Motion in Stress Myocardial Perfusion CT: Global and Regional Assessment. Clin. Imaging.

[29] Cui H., Liu C., Esworthy T., Huang Y., Yu Z.X., Zhou X., San H., Lee S.J., Hann S.Y., Boehm M. (2020). 4D Physiologically Adaptable Cardiac Patch: A 4-Month in Vivo Study for the Treatment of Myocardial Infarction. Sci. Adv..

[30] Miao S., Cui H., Nowicki M., Lee S.J., Almeida J., Zhou X., Zhu W., Yao X., Masood F., Plesniak M.W. (2018). Photolithographic-Stereolithographic-Tandem Fabrication of 4D Smart Scaffolds for Improved Stem Cell Cardiomyogenic Differentiation. Biofabrication.

[31] Wang Y., Cui H., Wang Y., Xu C., Esworthy T.J., Hann S.Y., Boehm M., Shen Y.L., Mei D., Zhang L.G. (2021). 4D Printed Cardiac Construct with Aligned Myofibers and Adjustable Curvature for Myocardial Regeneration. ACS Appl. Mater. Interfaces.

[32] Yu C., Ma X., Zhu W., Wang P., Miller K.L., Stupin J., Koroleva-Maharajh A., Hairabedian A., Chen S. (2019). Scanningless and Continuous 3D Bioprinting of Human Tissues with Decellularized Extracellular Matrix. Biomaterials.

[33] Liu J., Miller K., Ma X., Dewan S., Lawrence N., Whang G., Chung P., McCulloch A.D., Chen S. (2020). Direct 3D Bioprinting of Cardiac Micro-Tissues Mimicking Native Myocardium. Biomaterials.

[34] Grigoryan B., Paulsen S.J., Corbett D.C., Sazer D.W., Fortin C.L., Zaita A.J., Greenfield P.T., Calafat N.J., Gounley J.P., Ta A.H. (2019). Multivascular Networks and Functional Intravascular Topologies Within Biocompatible Hydrogels. Science.

[35] Tijore A., Irvine S.A., Sarig U., Mhaisalkar P., Baisane V., Venkatraman S. (2018). Contact Guidance for Cardiac Tissue Engineering Using 3D Bioprinted Gelatin Patterned Hydrogel. Biofabrication.

[36] Mehrotra S., Singh R.D., Bandyopadhyay A., Janani G., Dey S., Mandal B.B. (2021). Engineering Microsphere-Loaded Non-Mulberry Silk-Based 3D Bioprinted Vascularized Cardiac Patches with Oxygen-Releasing and Immunomodulatory Potential. ACS Appl. Mater. Interfaces.

[37] Maiullari F., Costantini M., Milan M., Pace V., Chirivì M., Maiullari S., Rainer A., Baci D., Marei H.E.S., Seliktar D. (2018). A Multi-Cellular 3D Bioprinting Approach for Vascularized Heart Tissue Engineering Based on HUVECs and IPSC-Derived Cardiomyocytes. Sci. Rep..

[38] Ahrens J.H., Uzel S.G.M., Skylar-Scott M., Mata M.M., Lu A., Kroll K.T., Lewis J.A. (2022). Programming Cellular Alignment in Engineered Cardiac Tissue via Bioprinting Anisotropic Organ Building Blocks. Adv. Mater..

[39] Wu H., Xu F., Jin H., Xue M., Zhang W., Yang J., Huang J., Jiang Y., Qiu B., Lin B. (2024). 3D Nanofiber-Assisted Embedded Extrusion Bioprinting for Oriented Cardiac Tissue Fabrication. ACS Biomater. Sci. Eng..

[40] Boularaoui S., Al Hussein G., Khan K.A., Christoforou N., Stefanini C. (2020). An Overview of Extrusion-Based Bioprinting with a Focus on Induced Shear Stress and Its Effect on Cell Viability. Bioprinting.

[41] Lee S., Sani E.S., Spencer A.R., Guan Y., Weiss A.S., Annabi N. (2020). Human-Recombinant-Elastin-Based Bioinks for 3D Bioprinting of Vascularized Soft Tissues. Adv. Mater..

[42] McCormack A., Highley C.B., Leslie N.R., Melchels F.P.W. (2020). 3D Printing in Suspension Baths: Keeping the Promises of Bioprinting Afloat. Trends Biotechnol..

[43] Lee A., Hudson A.R., Shiwarski D.J., Tashman J.W., Hinton T.J., Yerneni S., Bliley J.M., Campbell P.G., Feinberg A.W. (2019). 3D Bioprinting of Collagen to Rebuild Components of the Human Heart. Science.

[44] Fang Y., Guo Y., Wu B., Liu Z., Ye M., Xu Y., Ji M., Chen L., Lu B., Nie K. (2023). Expanding Embedded 3D Bioprinting Capability for Engineering Complex Organs with Freeform Vascular Networks. Adv. Mater..

[45] Shiwarski D.J., Hudson A.R., Tashman J.W., Feinberg A.W. (2021). Emergence of FRESH 3D Printing as a Platform for Advanced Tissue Biofabrication. APL Bioeng..

[46] Koti P., Muselimyan N., Mirdamadi E., Asfour H., Sarvazyan N.A. (2019). Use of GelMA for 3D Printing of Cardiac Myocytes and Fibroblasts. J. 3D Print. Med..

[47] Bonatti A.F., Vozzi G., Chua C.K., De Maria C. (2022). A Deep Learning Quality Control Loop of the Extrusion-Based Bioprinting Process. Int. J. Bioprint.

[48] Sergis V., Kelly D., Pramanick A., Britchfield G., Mason K., Daly A.C. (2025). In-Situ Quality Monitoring During Embedded Bioprinting Using Integrated Microscopy and Classical Computer Vision. Biofabrication.

[49] Bernal P.N., Bouwmeester M., Madrid-Wolff J., Falandt M., Florczak S., Rodriguez N.G., Li Y., Größbacher G., Samsom R.A., van Wolferen M. (2022). Volumetric Bioprinting of Organoids and Optically Tuned Hydrogels to Build Liver-like Metabolic Biofactories. Adv. Mater..

[50] Lian L., Xie M., Luo Z., Zhang Z., Maharjan S., Mu X., Garciamendez-Mijares C.E., Kuang X., Sahoo J.K., Tang G. (2024). Rapid Volumetric Bioprinting of Decellularized Extracellular Matrix Bioinks. Adv. Mater..

[51] Jones L.S., Filippi M., Michelis M.Y., Balciunaite A., Yasa O., Aviel G., Narciso M., Freedrich S., Generali M., Tzahor E. (2024). Multidirectional Filamented Light Biofabrication Creates Aligned and Contractile Cardiac Tissues. Adv. Sci..

[52] Ribezzi D., Zegwaart J.P., Van Gansbeke T., Tejo-Otero A., Florczak S., Aerts J., Delrot P., Hierholzer A., Fussenegger M., Malda J. (2025). Multi-Material Volumetric Bioprinting and Plug-and-Play Suspension Bath Biofabrication via Bioresin Molecular Weight Tuning and via Multiwavelength Alignment Optics. Adv. Mater..

[53] Mousavi A., Hedayatnia A., van Vliet P.P., Dartora D.R., Wong N., Rafatian N., Nuyt A.M., Moraes C., Ajji A., Andelfinger G. (2024). Development of Photocrosslinkable Bioinks with Improved Electromechanical Properties for 3D Bioprinting of Cardiac BioRings. Appl. Mater. Today.

[54] Groll J., Burdick J.A., Cho D.W., Derby B., Gelinsky M., Heilshorn S.C., Jüngst T., Malda J., Mironov V.A., Nakayama K. (2018). A Definition of Bioinks and Their Distinction from Biomaterial Inks. Biofabrication.

[55] Chopin-Doroteo M., Mandujano-Tinoco E.A., Krötzsch E. (2021). Tailoring of the Rheological Properties of Bioinks to Improve Bioprinting and Bioassembly for Tissue Replacement. Biochim. Biophys. Acta (BBA)—Gen. Subj..

[56] Shin Y.J., Shafranek R.T., Tsui J.H., Walcott J., Nelson A., Kim D.H. (2021). 3D Bioprinting of Mechanically Tuned Bioinks Derived from Cardiac Decellularized Extracellular Matrix. Acta Biomater..

[57] Diamantides N., Wang L., Pruiksma T., Siemiatkoski J., Dugopolski C., Shortkroff S., Kennedy S., Bonassar L.J. (2017). Correlating Rheological Properties and Printability of Collagen Bioinks: The Effects of Riboflavin Photocrosslinking and PH. Biofabrication.

[58] Basara G., Ozcebe S.G., Ellis B.W., Zorlutuna P. (2021). Tunable Human Myocardium Derived Decellularized Extracellular Matrix for 3D Bioprinting and Cardiac Tissue Engineering. Gels.

[59] Stola G.P., Paoletti C., Nicoletti L., Paul G., Cassino C., Marchese L., Chiono V., Marcello E. (2024). Internally-Crosslinked Alginate Dialdehyde/Alginate/Gelatin-Based Hydrogels as Bioinks for Prospective Cardiac Tissue Engineering Applications. Int. J. Bioprinting.

[60] Ketabat F., Maris T., Duan X., Yazdanpanah Z., Kelly M.E., Badea I., Chen X. (2023). Optimization of 3D Printing and In Vitro Characterization of Alginate/Gelatin Lattice and Angular Scaffolds for Potential Cardiac Tissue Engineering. Front. Bioeng. Biotechnol..

[61] Jalilinejad N., Rabiee M., Baheiraei N., Ghahremanzadeh R., Salarian R., Rabiee N., Akhavan O., Zarrintaj P., Hejna A., Saeb M.R. (2023). Electrically Conductive Carbon-Based (Bio)-Nanomaterials for Cardiac Tissue Engineering. Bioeng. Transl. Med..

[62] Tsui J.H., Leonard A., Camp N.D., Long J.T., Nawas Z.Y., Chavanachat R., Smith A.S.T., Choi J.S., Dong Z., Ahn E.H. (2021). Tunable Electroconductive Decellularized Extracellular Matrix Hydrogels for Engineering Human Cardiac Microphysiological Systems. Biomaterials.

[63] Basara G., Saeidi-Javash M., Ren X., Bahcecioglu G., Wyatt B.C., Anasori B., Zhang Y., Zorlutuna P. (2022). Electrically Conductive 3D Printed Ti3C2Tx MXene-PEG Composite Constructs for Cardiac Tissue Engineering. Acta Biomater..

[64] de Oliveira M., Pereira C., Calmeiro T., Jana S., Fernandes S., Inácio J., Belo J., Matela N., Borges J.P., Fortunato E. (2025). Biofabricated Electroactive Hydrogel Platforms for Cardiac Cell Stimulus with Alginate-Gelatin-Cnt/Mxene Matrices. Mxene Matrices.

[65] Zhu K., Shin S.R., van Kempen T., Li Y.C., Ponraj V., Nasajpour A., Mandla S., Hu N., Liu X., Leijten J. (2017). Gold Nanocomposite Bioink for Printing 3D Cardiac Constructs. Adv. Funct. Mater..

[66] Ramirez S.P., Hernandez I., Balcorta H.V., Kumar P., Kumar V., Poon W., Joddar B. (2024). Microcomputed Tomography for the Microstructure Evaluation of 3D Bioprinted Scaffolds. ACS Appl. Bio Mater..

[67] Testore D., Zoso A., Kortaberria G., Sangermano M., Chiono V. (2022). Electroconductive Photo-Curable PEGDA-Gelatin/PEDOT:PSS Hydrogels for Prospective Cardiac Tissue Engineering Application. Front. Bioeng. Biotechnol..

[68] Roshanbinfar K., Schiffer M., Carls E., Angeloni M., Koleśnik-Gray M., Schruefer S., Schubert D.W., Ferrazzi F., Krstić V., Fleischmann B.K. (2024). Electrically Conductive Collagen-PEDOT:PSS Hydrogel Prevents Post-Infarct Cardiac Arrhythmia and Supports HiPSC-Cardiomyocyte Function. Adv. Mater..

[69] Gionet-Gonzales M., Gathman G., Rosas J., Kunisaki K.Y., Inocencio D.G.P., Hakami N., Milburn G.N., Pitenis A.A., Campbell K.S., Pruitt B.L. (2024). Stress Relaxation Rates of Myocardium from Failing and Non-Failing Hearts. bioRxiv.

[70] Wang J., Gao H., Hu Y., Zhang N., Zhou W., Wang C., Binks B.P., Yang Z. (2021). 3D Printing of Pickering Emulsion Inks to Construct Poly(D,L-lactide-co-trimethylene carbonate)-Based Porous Bioactive Scaffolds with Shape Memory Effect. J. Mater. Sci..

[71] Cui H., Miao S., Esworthy T., Lee S.J., Zhou X., Hann S.Y., Webster T.J., Harris B.T., Zhang L.G. (2019). A Novel Near-Infrared Light Responsive 4D Printed Nanoarchitecture with Dynamically and Remotely Controllable Transformation. Nano Res..

[72] Adams S.D., Ashok A., Kanwar R.K., Kanwar J.R., Kouzani A.Z. (2017). Integrated 3D Printed Scaffolds and Electrical Stimulation for Enhancing Primary Human Cardiomyocyte Cultures. Bioprinting.

[73] Zhou B., Shi X., Tang X., Zhao Q., Wang L., Yao F., Hou Y., Wang X., Feng W., Wang L. (2022). Functional Isolation, Culture and Cryopreservation of Adult Human Primary Cardiomyocytes. Signal Transduct. Target. Ther..

[74] Wang Z., Lee S.J., Cheng H.J., Yoo J.J., Atala A. (2018). 3D Bioprinted Functional and Contractile Cardiac Tissue Constructs. Acta Biomater..

[75] Das S., Kim S.W., Choi Y.J., Lee S., Lee S.H., Kong J.S., Park H.J., Cho D.W., Jang J. (2019). Decellularized Extracellular Matrix Bioinks and the External Stimuli to Enhance Cardiac Tissue Development In Vitro. Acta Biomater..

[76] Tang J., Cui X., Caranasos T.G., Hensley M.T., Vandergriff A.C., Hartanto Y., Shen D., Zhang H., Zhang J., Cheng K. (2017). Heart Repair Using Nanogel-Encapsulated Human Cardiac Stem Cells in Mice and Pigs with Myocardial Infarction. ACS Nano.

[77] Du B., Dai Z., Wang H., Ren Z., Li D. (2025). Advances and Prospects in Using Induced Pluripotent Stem Cells for 3D Bioprinting in Cardiac Tissue Engineering. Rev. Cardiovasc. Med..

[78] Karbassi E., Fenix A., Marchiano S., Muraoka N., Nakamura K., Yang X., Murry C.E. (2020). Cardiomyocyte Maturation: Advances in Knowledge and Implications for Regenerative Medicine. Nat. Rev. Cardiol..

[79] Dhahri W., Sadikov Valdman T., Wilkinson D., Pereira E., Ceylan E., Andharia N., Qiang B., Masoudpour H., Wulkan F., Quesnel E. (2022). In Vitro Matured Human Pluripotent Stem Cell-Derived Cardiomyocytes Form Grafts with Enhanced Structure and Function in Injured Hearts. Circulation.

[80] Lundy S.D., Zhu W.Z., Regnier M., Laflamme M.A. (2013). Structural and Functional Maturation of Cardiomyocytes Derived from Human Pluripotent Stem Cells. Stem Cells Dev..

[81] Piccini I., Rao J., Seebohm G., Greber B. (2015). Human Pluripotent Stem Cell-Derived Cardiomyocytes: Genome-Wide Expression Profiling of Long-Term In Vitro Maturation in Comparison to Human Heart Tissue. Genom. Data.

[82] Tiburcy M., Hudson J.E., Balfanz P., Schlick S., Meyer T., Liao M.L.C., Levent E., Raad F., Zeidler S., Wingender E. (2017). Defined Engineered Human Myocardium with Advanced Maturation for Applications in Heart Failure Modeling and Repair. Circulation.

[83] Ronaldson-Bouchard K., Ma S.P., Yeager K., Chen T., Song L., Sirabella D., Morikawa K., Teles D., Yazawa M., Vunjak-Novakovic G. (2018). Advanced Maturation of Human Cardiac Tissue Grown from Pluripotent Stem Cells. Nature.

[84] Ellis M.E., Harris B.N., Hashemi M., Harvell B.J., Bush M.Z., Hicks E.E., Finklea F.B., Wang E.M., Nataraj R., Young N.P. (2022). Human Induced Pluripotent Stem Cell Encapsulation Geometry Impacts Three-Dimensional Developing Human Engineered Cardiac Tissue Functionality. Tissue Eng. Part A.

[85] Kupfer M.E., Lin W.H., Ravikumar V., Qiu K., Wang L., Gao L., Bhuiyan D.B., Lenz M., Ai J., Mahutga R.R. (2020). In Situ Expansion, Differentiation, and Electromechanical Coupling of Human Cardiac Muscle in a 3D Bioprinted, Chambered Organoid. Circ. Res..

[86] Deidda V., Ventisette I., Langione M., Giammarino L., Pioner J.M., Credi C., Carpi F. (2024). 3D-Printable Gelatin Methacrylate-Xanthan Gum Hydrogel Bioink Enabling Human Induced Pluripotent Stem Cell Differentiation into Cardiomyocytes. J. Funct. Biomater..

[87] Anil Kumar S., Alonzo M., Allen S.C., Abelseth L., Thakur V., Akimoto J., Ito Y., Willerth S.M., Suggs L., Chattopadhyay M. (2019). A Visible Light-Cross-Linkable, Fibrin-Gelatin-Based Bioprinted Construct with Human Cardiomyocytes and Fibroblasts. ACS Biomater. Sci. Eng..

[88] Khoury R.E., Nagiah N., Mudloff J.A., Thakur V., Chattopadhyay M., Joddar B. (2021). 3D Bioprinted Spheroidal Droplets for Engineering the Heterocellular Coupling Between Cardiomyocytes and Cardiac Fibroblasts. Cyborg Bionic Syst..

[89] Howard C.M., Baudino T.A. (2014). Dynamic Cell-Cell and Cell-ECM Interactions in the Heart. J. Mol. Cell Cardiol..

[90] Cofiño-Fabres C., Boonen T., Rivera-Arbeláez J.M., Rijpkema M., Blauw L., Rensen P.C.N., Schwach V., Ribeiro M.C., Passier R., Cofiño-Fabres C. (2024). Micro-Engineered Heart Tissues On-Chip with Heterotypic Cell Composition Display Self-Organization and Improved Cardiac Function. Adv. Healthc. Mater..

[91] Brady E.L., Prado O., Johansson F., Mitchell S.N., Martinson A.M., Karbassi E., Reinecke H., Murry C.E., Davis J., Stevens K.R. (2023). Engineered Tissue Vascularization and Engraftment Depends on Host Model. Sci. Rep..

[92] Han K., He J., Fu L., Mao M., Kang Y., Li D. (2022). Engineering Highly-Aligned Three-Dimensional (3D) Cardiac Constructs for Enhanced Myocardial Infarction Repair. Biofabrication.

[93] Roche C.D., Lin H., Huang Y., de Bock C.E., Beck D., Xue M., Gentile C. (2023). 3D Bioprinted Alginate-Gelatin Hydrogel Patches Containing Cardiac Spheroids Recover Heart Function in a Mouse Model of Myocardial Infarction. Bioprinting.

[94] Jones L.S., Rodriguez H., Biefer C., Mekkattu M., Thijssen Q., Amicone A., Bock A., Weisskopf M., Zorndt D., Meier D. (2025). Volumetric 3D Printing and Melt-Electrowriting to Fabricate Implantable Reinforced Cardiac Tissue Patches. Adv. Mater..

[95] Guesdon R., Santoro S., Cras A., Pagin E., Serteyn D., Ceusters J., Guillemot F., Hagège A., Menasché P. (2025). Repair of Infarcted Myocardium by Skeletal Muscle-Derived Mesenchymal Stromal Cells Delivered by a Bioprinted Collagen Patch. Stem Cell Res. Ther..

[96] Wang Z., Qin C., Liao Z., Zhang H., Lu H., Xiao Y., Wu C. (2025). Inorganic Biomaterials Inducing Scaffolds Pre-Neuralization for Infarcted Myocardium Repair. Adv. Mater..

[97] Asulin M., Michael I., Shapira A., Dvir T. (2021). One-Step 3D Printing of Heart Patches with Built-In Electronics for Performance Regulation. Adv. Sci..

[98] Bar A., Kryukov O., Etzion S., Cohen S. (2023). Engineered Extracellular Vesicle-Mediated Delivery of MiR-199a-3p Increases the Viability of 3D-Printed Cardiac Patches. Int. J. Bioprint.

[99] Gil C.J., Allphin A.J., Jin L., Amoli M.S., Rezapourdamanab S., Tomov M.L., Hwang B., Sridhar V., El Shammas L.R., Wu Y. (2025). Image-Guided Cardiac Regeneration via a 3D Bioprinted Vascular Patch with Built-in CT Visibility. Chem. Eng. J..

[100] Jebran A.F., Seidler T., Tiburcy M., Daskalaki M., Kutschka I., Fujita B., Ensminger S., Bremmer F., Moussavi A., Yang H. (2025). Engineered Heart Muscle Allografts for Heart Repair in Primates and Humans. Nature.

[101] Lu K. (2023). Maturation of Human Induced Pluripotent Stem Cell Based Myocardium by Biomechanical Stimulation of Three-Dimensional Tissue Cultures. Ph.D. Thesis.

[102] Dellaquila A., Le Bao C., Letourneur D., Simon-Yarza T., Dellaquila A., Le Bao C., Letourneur D., Simon-Yarza T., Dellaquila Biomolecular Photonics A. (2021). In Vitro Strategies to Vascularize 3D Physiologically Relevant Models. Adv. Sci..

[103] Maihemuti W., Murata K., Abulaiti M., Minatoya K., Masumoto H. (2024). Simultaneous Electro-Dynamic Stimulation Accelerates Maturation of Engineered Cardiac Tissues Generated by Human IPS Cells. Biochem. Biophys. Res. Commun..

[104] Ershad F., Rao Z., Maharajan S., Mesquita F.C.P., Ha J., Gonzalez L., Haideri T., Da Costa E.C., Moctezuma-Ramirez A., Wang Y. (2025). Bioprinted Optoelectronically Active Cardiac Tissues. Sci. Adv..

[105] Schwach V., Passier R. (2019). Native Cardiac Environment and Its Impact on Engineering Cardiac Tissue. Biomater. Sci..

[106] DeLaughter D.M., Bick A.G., Wakimoto H., McKean D., Gorham J.M., Kathiriya I.S., Hinson J.T., Homsy J., Gray J., Pu W. (2016). Single-Cell Resolution of Temporal Gene Expression during Heart Development. Dev. Cell.

[107] Quaife-Ryan G.A., Sim C.B., Ziemann M., Kaspi A., Rafehi H., Ramialison M., El-Osta A., Hudson J.E., Porrello E.R. (2017). Multicellular Transcriptional Analysis of Mammalian Heart Regeneration. Circulation.

[108] Alonzo M., El Khoury R., Nagiah N., Thakur V., Chattopadhyay M., Joddar B. (2022). 3D Biofabrication of a Cardiac Tissue Construct for Sustained Longevity and Function. ACS Appl. Mater. Interfaces.

[109] Maas R.G.C., Beekink T., Chirico N., Snijders Blok C.J.B., Dokter I., Sampaio-Pinto V., van Mil A., Doevendans P.A., Buikema J.W., Sluijter J.P.G. (2023). Generation, High-Throughput Screening, and Biobanking of Human-Induced Pluripotent Stem Cell-Derived Cardiac Spheroids. J. Vis. Exp..

[110] Daly A.C., Davidson M.D., Burdick J.A. (2021). 3D Bioprinting of High Cell-Density Heterogeneous Tissue Models through Spheroid Fusion within Self-Healing Hydrogels. Nat. Commun..

[111] Basara G., Celebi L.E., Ronan G., Discua Santos V., Zorlutuna P. (2024). 3D Bioprinted Aged Human Post-Infarct Myocardium Tissue Model. Health Sci. Rep..

[112] Miller K.L., Sit I., Xiang Y., Wu J., Pustelnik J., Tang M., Kiratitanaporn W., Grassian V., Chen S. (2023). Evaluation of CuO Nanoparticle Toxicity on 3D Bioprinted Human IPSC-Derived Cardiac Tissues. Bioprinting.

[113] Hwang D.G., Choi H., Yong U., Kim D., Kang W., Park S.M., Jang J. (2024). Bioprinting-Assisted Tissue Assembly for Structural and Functional Modulation of Engineered Heart Tissue Mimicking Left Ventricular Myocardial Fiber Orientation. Adv. Mater..

[114] Zeng Q., Yang Y., Wang H., Ye T., Wang Z., Chai M., Lu Z., He S., Yang H., Zhang J.Z. (2026). 3D Printing of Structural Bionic and Functionalized Hydrogels for the Construction of Macroscale Human Cardiac Tissues. Biomaterials.

[115] Bliley J.M., Stang M.A., Behre A., Feinberg A.W. (2024). Advances in 3D Bioprinted Cardiac Tissue Using Stem Cell-Derived Cardiomyocytes. Stem Cells Transl. Med..

[116] Ho D.L.L., Lee S., Du J., Weiss J.D., Tam T., Sinha S., Klinger D., Devine S., Hamfeldt A., Leng H.T. (2022). Large-Scale Production of Wholly Cellular Bioinks via the Optimization of Human Induced Pluripotent Stem Cell Aggregate Culture in Automated Bioreactors. Adv. Healthc. Mater..

[117] da Silva V.A., Leung M.C., Clayton M., Oommen L., Madrigal H., Laksman Z., Yu B., Willerth S.M. (2025). Building the Framework for Bioprinted Human Heart Tissue: Recent Developments and Future Prospects. Regen. Med..

